# Interaction of the Atypical Tetracyclines Chelocardin and Amidochelocardin with Renal Drug Transporters

**DOI:** 10.1021/acsptsci.4c00183

**Published:** 2024-06-11

**Authors:** Katharina Rox, Annett Kühne, Jennifer Herrmann, Rolf Jansen, Stephan Hüttel, Steffen Bernecker, Yohannes Hagos, Mark Brönstrup, Marc Stadler, Thomas Hesterkamp, Rolf Müller

**Affiliations:** † Department of Chemical Biology, Helmholtz Centre for Infection Research (HZI), 38124 Braunschweig, Germany; ‡ German Center for Infection Research (DZIF), partner site Braunschweig-Hannover, 38124 Braunschweig, Germany; § PortaCellTec Biosciences GmbH, 37079 Göttingen, Germany; ∥ Department of Microbial Natural Products, Helmholtz Institute for Pharmaceutical Research Saarland (HIPS), Helmholtz Centre for Infection Research (HZI) and Department of Pharmacy, Saarland University, 66123 Saarbrücken, Germany; ⊥ Department of Microbial Drugs, Helmholtz Centre for Infection Research (HZI), 38124 Braunschweig, Germany; # Translational Product Management Office, German Center for Infection Research (DZIF), partner site Braunschweig-Hannover, 38124 Braunschweig, Germany

**Keywords:** tetracycline, chelocardin, amidochelocardin, organic anion transporter, efflux transporter, p-gp, organic cation transporter, Michaelis−Menten, drug transporter, kidney, inhibitor

## Abstract

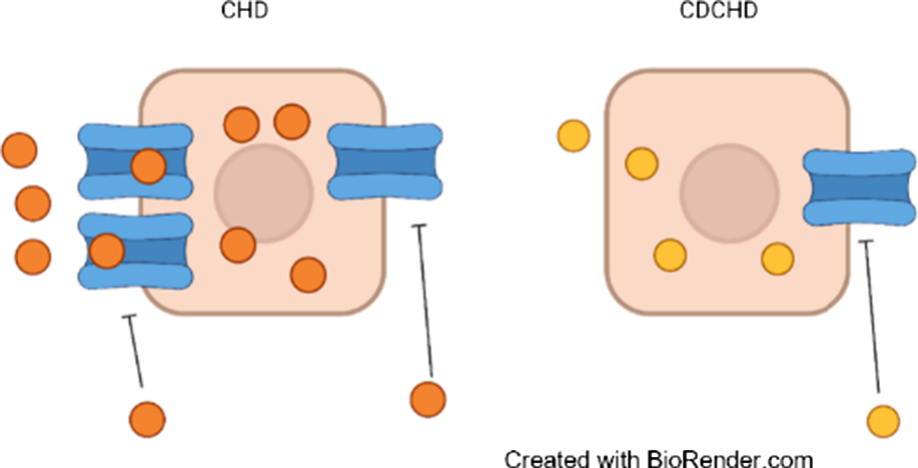

Antimicrobial resistance is expected to increase mortality rates by up to several million deaths per year by 2050 without new treatment options at hand. Recently, we characterized the pharmacokinetic (PK) and pharmacodynamic properties of two atypical tetracyclines, chelocardin (CHD) and amidochelocardin (CDCHD) that exhibit no cross-resistance with clinically used antibacterials. Both compounds were preferentially renally cleared and demonstrated pronounced effects in an ascending urinary tract infection model against *E. coli*. Renal drug transporters are known to influence clearance into the urine. In particular, inhibition of apical transporters in renal tubular epithelial cells can lead to intracellular accumulation and potential cell toxicity, whereas inhibition of basolateral transporters can cause a higher systemic exposure. Here, selected murine and human organic cation (Oct), organic anion (Oat), and efflux transporters were studied to elucidate interactions with CHD and CDCHD underlying their PK behavior. CHD exhibited stronger inhibitory effects on mOat1 and mOat3 and their human homologues hOAT1 and hOAT3 compared to CDCHD. While CHD was a substrate of mOat3 and mOct1, CDCHD was not. By contrast, no inhibitory effect was observed on Octs. CDCHD rather appeared to foster enhanced substrate transport on mOct1. CHD and CDCHD inhibited the efflux transporter hMRP2 on the apical side. In summary, the substrate nature of CHD in conjunction with its autoinhibition toward mOat3 rationalizes the distinct urine concentration profile compared to CDCHD that was previously observed in vivo. Further studies are needed to investigate the accumulation in renal tubular cells and the nephrotoxicity risk.

There is a tremendous rise in antimicrobial resistance (AMR) over the last decades with an expected increase in the number of fatalities by 2050.^1−3^ Hence, there is a high demand for novel treatment options.^4,5^ Tetracyclines are a class of broad-spectrum antibacterials. Doxycycline is currently used to treat intracellular, neurological as well as sexually transmitted infections first line and pneumonia as second line.^6−8^ However, the use has partially declined due to clinical resistance.^9,10^ The same applies to minocycline, a second generation of tetracycline. However, also the newest, third-generation tetracyclines, such as tigecycline, are starting to face resistance problems.^11−15^ This might not yet be widely seen for the most recent ones, such as eravacycline or omadacycline, but is expected to augment as well.^16−19^

Few years ago, the atypical tetracycline, chelocardin (CHD), was rediscovered.^20−23^ CHD was under clinical investigation up to a phase II clinical trial against urinary tract infections but was abandoned despite signs of efficacy.^20,24^ No further information could be retrieved to understand why the clinical development of CHD had been stopped. Based on CHD, amidochelocardin (CDCHD) was developed via biosynthetic engineering with the aim to combine favorable chemical features known from the tetracycline SAR.^22,23,25^ Similar to tetracyclines, CDCHD harbors a carboxamido function in the side chain of ring A, whereas CHD does not (Figure 1).^23,26^ CDCHD indeed overcomes resistance mechanisms typically observed for tetracyclines as well as for CHD. Hence, it exhibits resistance-breaking properties.^27,28^ Recently, we have performed a pharmacokinetic (PK) and pharmacodynamic (PD) evaluation of CHD and CDCHD. Therein, both atypical tetracyclines, CHD and CDCHD, showed pronounced effects in an ascending urinary tract infection model against uropathogenic *E. coli*.^29^ We have shown previously that after administration of 15 mg/kg IV, high concentrations of the parent compound were found in urine for CHD and CDCHD. Urine levels for CHD were found to be 10-fold higher compared to that for CDCHD but decreased more rapidly.^29^

**Figure 1:**
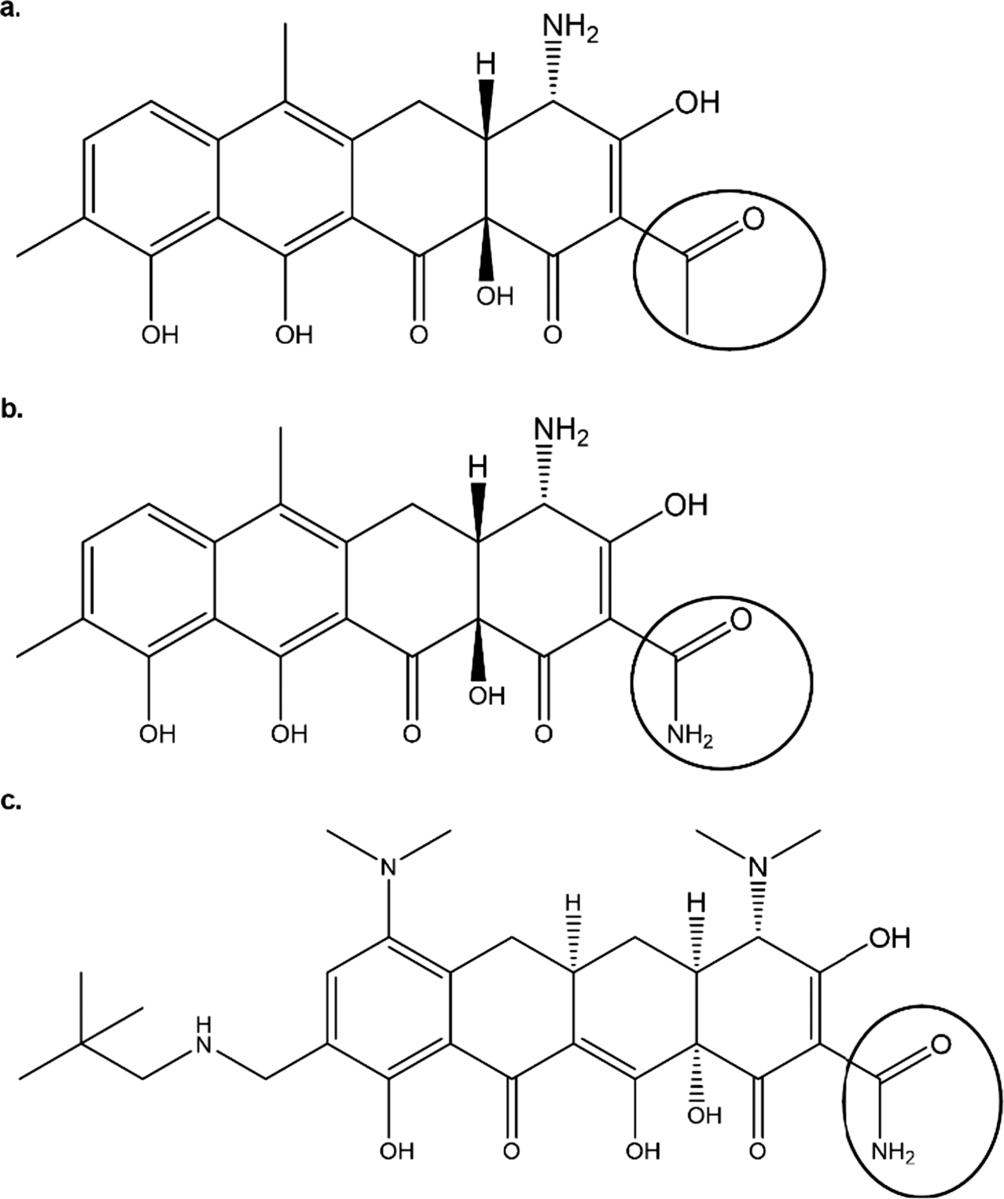
Chemical structures of CHD (a), CDCHD (b), and omadacycline (c). The differences in the carbamid group are highlighted.

Renal drug transporters play an important role for the clearance of drugs into urine and are implicated in drug–drug interactions.^30−33^ The most prominent family of transporters is the solute carrier (SLC) superfamily 22 (SLC22). Within this family, organic anion transporters (OATs) and organic cation transporters (OCTs) are represented (Figure 2).^34−39^ Whereas some transporters are exclusively located in the basolateral membrane of renal tubular epithelial cells, such as hOAT1/mOat1 and hOAT3/mOat3, or in the apical membrane (hOAT4), the location of others depends on the species as, e.g., the hOAT2 transporter is located in the basolateral membrane and the mOat2 transporter in the apical membrane. Importantly, mOat2 is responsible for drug re-absorption from the lumen, whereas hOAT2 is responsible for drug secretion into the proximal tubule cell.^31,35,40^ Depending on the affinity of the substrate, hOAT4 can either contribute to efflux or re-absorption of substrates into the proximal tubule epithelial cell.^41^ It is known that drugs have different affinities to rodent and human transporters.^42^ Moreover, the multidrug resistance-associated protein 2 (MRP2), also known as canalicular multispecific organic anion transporter (cMOAT), is an efflux transporter, located at the apical membrane and responsible for the excretion of xenobiotics into bile or urine.^43−45^ Other important families include the multidrug and toxin extrusion transporters MATE, belonging to the SLC47 family. These are mainly located on the apical side of the epithelia. Whereas MATE2-K is exclusively located in the kidneys, MATE1 is also found in other tissues, such as liver or musculoskeletal tissue.^46,47^ For MATE1, no major differences regarding transport have been observed between rodents and humans.^48^ Another important transporter is multidrug resistance protein 1 (MDR1 or p-glycoprotein (p-gp)), which is an efflux transporter located at the luminal membrane of proximal tubule cells. It is responsible for excreting compounds from the kidney into urine and is not only found in kidneys but also in other polarized epithelia, such as in liver and brain.^33,49^ In mice, two subtypes, mMdr1a and mMdr1b, exist, both of which transport xenobiotics.^50^

**Figure 2:**
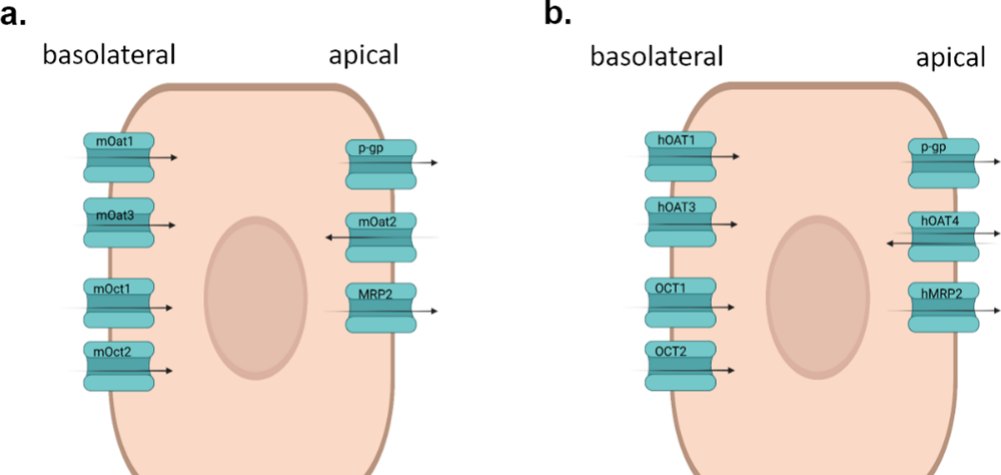
Localization of murine and human transporters in kidney tubular cells. The localization of murine (a) and human (b) renal drug transporters in tubular cells is depicted. The arrows show the direction of drug transport. In the case of hOAT4 it can serve as an efflux transporter or transporter causing reabsorption. Created with BioRender.com.

As several renal drug transporters play a role in the clearance of drugs into urine^30,31^ and as CHD and CDCHD parental compounds reached high concentrations in urine,^29^ the aim of this study was to find out whether CHD and CDCHD are potential substrates or inhibitors of transporters. This could, in principle, lead to accumulation in the kidneys and subsequent nephrotoxic side effects.

We observed that CHD, but not CDCHD, inhibited mOat1 and mOat3 as well as their human homologues in a concentration-dependent manner on the basolateral side. In addition, we show that CHD was a substrate of mOat3, whereas that does not apply to CDCHD. Moreover, we demonstrate that CHD and CDCHD inhibited hMRP2, an efflux transporter on the apical side. Thus, this study provides a first insight into renal transporter inhibition signatures of CHD and CDCHD, thereby providing a data basis for assessing the accumulation risk of CHD and CDCHD.

## Results

### CHD Shows a Different Inhibition Profile on Organic Anion Transporters than CDCHD

First, we assessed the effects of CHD and CDCHD on selected mouse and human organic anion transporters in cell lines stably overexpressing the respective transporter, i.e., mOat1, mOat2, and mOat3 (murine transporters) and hOAT1, hOAT3, hOAT4, and hMRP2 (human transporters). mOat1, mOat3, hOAT1, and hOAT3 are located on the basolateral side, whereas mOat2, hOAT4, and hMRP2 are located on the apical side of polarized epithelia.^35,40,43^

In these assays, we aimed to determine the uptake ratio as well as potential inhibitory effects of CHD and CDCHD. Consequently, the uptake of a transporter-specific probe substrate in the presence of two different concentrations of CHD and CDCHD, either 1 or 10 μM, was measured (Figure S1). Additionally, a specific known probe inhibitor of the respective transporter was used as a comparator, i.e., probenecid for mOat1/hOAT1 and mOat3/hOAT3^51^, indomethacin for mOat2,^42^ and benzbromarone for hMRP2^42^. For hOAT4, sulfobromophtalein (BSP) was used as an inhibitor.

**Figure 3:**
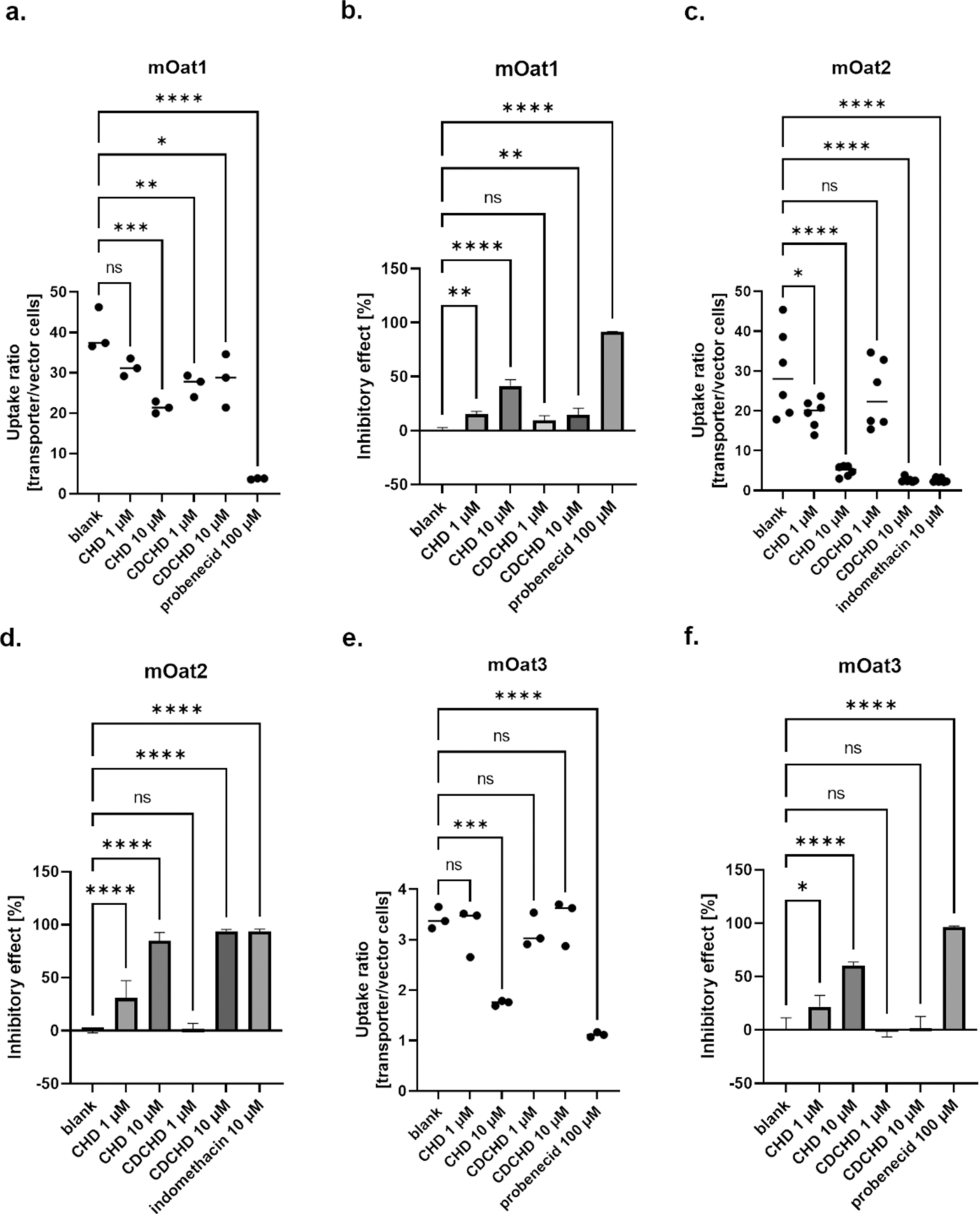
Inhibitory effects on murine organic anion transporters. The uptake ratio and the inhibitory effect were determined for mOat1 (a, b), mOat2 (c, d), and mOat3 (e, f). The uptake ratio of the substrate as a function of amount per transporter/amount per vector cells was calculated for each condition (a, c, e): untreated, addition of CHD at 1 and 10 μM, addition of CDCHD at 1 and 10 μM, and addition of inhibitor. The inhibitory effect as a net uptake compared to total net uptake of blank cells in percent was calculated for each condition (b, d, f): untreated, addition of CHD at 1 and 10 μM, addition of CDCHD at 1 and 10 μM, and addition of inhibitor. Both CHD and CDCHD decreased the uptake ratio into mOat1-overexpressing cells, whereas CHD decreased the uptake ratio in a concentration-dependent manner (*n* = 3 per condition) (a). CHD and CDCHD showed an inhibitory effect on mOat1. For CHD, the inhibitory effect was concentration-dependent. The inhibitory effect at 10 μM CHD was around 50%, half of the maximum effect achieved with probenecid (*n* = 3 per condition) (b). CHD and CDCHD decreased the uptake ratio into mOat2-overexpressing cells in a concentration-dependent manner (*n* = 6 per condition) (c). CHD and CDCHD showed an inhibitory effect on mOat2. The effect at 10 μM of CHD or CDCHD was in the same range as seen for indomethacin (*n* = 6 per condition) (d). Only CHD decreased the uptake ratio into mOat3-overexpressing cells in a concentration-dependent manner, whereas no effect was seen for CDCHD (*n* = 3 per condition) (e). CHD showed an inhibitory effect on mOat3. For CHD, the inhibitory effect was concentration-dependent. The inhibitory effect at 10 μM CHD was around 50%, half of the maximum effect achieved with probenecid (*n* = 3 per condition) (f). Statistical testing was performed using an ordinary one-way ANOVA. ns, not significant; *: *p* < 0.05; ** : *p* < 0.01; ***: *p* < 0.001; ****: *p* < 0.0001.

For the mOat1 transporter, a concentration-dependent decreased uptake upon the addition of CHD was observed. Albeit decreased uptake, this inhibition was not concentration-dependent in the case of CDCHD (Figures 3a, and S2a). In addition, CHD only caused a slight concentration-dependent inhibitory effect of less than 50 %, which was not in the range of the comparator probenecid (Figure 3b). By contrast, for mOat2, a concentration-dependent decreased uptake ratio was seen for both atypical tetracyclines, CHD and CDCHD. At the concentration of 10 μM, this was in the same range as detected for the probe inhibitor indomethacin (Figures 3c, and S2b). The same was observed when calculating the inhibitory effect, which was nearly 100 % at 10 μM for CHD and CDCHD (Figure 3d). Next, we determined the uptake ratio for mOat3. Here, a significantly decreased uptake was observed in the presence of 10 μM CHD. However, this was not as strong as seen for probenecid. No effects on uptake were observed for CDCHD at both tested concentrations (Figures 3e, and S2c). When calculating the inhibitory effect, a concentration dependency was observed for CHD with >50 % inhibition at 10 μM. By contrast, no inhibitory effect was determined for CDCHD (Figure 3f).

**Figure 4:**
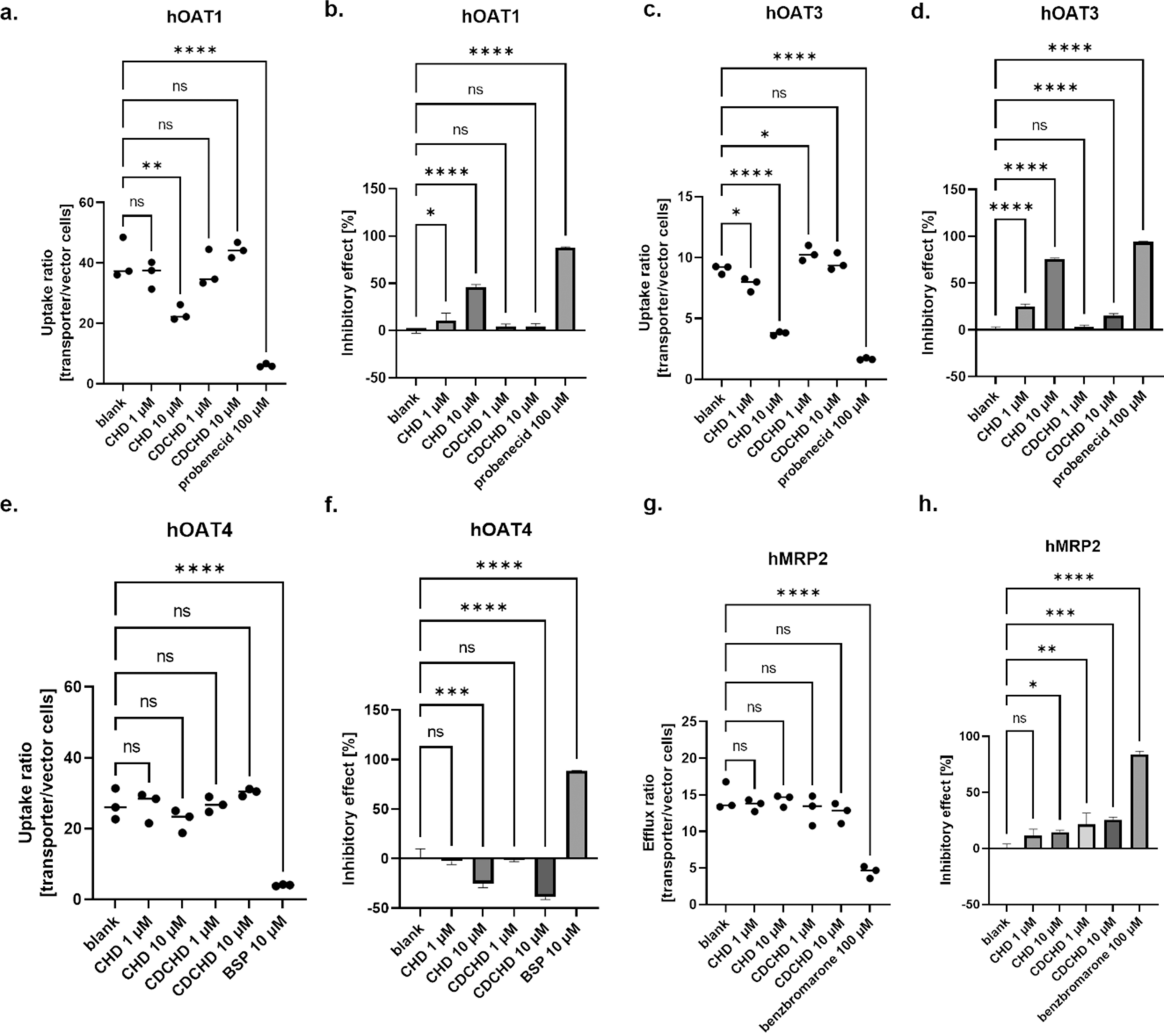
Interactions of CHD and CDCHD with human organic anion transporters. The uptake ratio and the inhibitory effect were determined for hOAT1 (a, b), hOAT3 (c, d), hOAT4 (e, f), and hMRP2 (g, h). The uptake ratio of substrate as a function of amount per transporter/amount per vector cells was calculated for each condition (a, c, e): untreated, addition of CHD at 1 and 10 μM, addition of CDCHD at 1 and 10 μM, and addition of inhibitor. The inhibitory effect as a net uptake compared to total net uptake of blank cells in percent was calculated for each condition (b, d, f, h): untreated, addition of CHD at 1 and 10 μM, addition of CDCHD at 1 and 10 μM, and addition of inhibitor. Moreover, the efflux ratio of substrate as a function of amount per transporter/amount per vector cells was calculated for each condition (g). Only CHD decreased the uptake ratio into hOAT1-overexpressing cells in a concentration-dependent manner (*n* = 3 per condition) (a). Only CHD showed an inhibitory effect on hOAT1. For CHD, the inhibitory effect was concentration-dependent. The inhibitory effect at 10 μM CHD was around 50%, half of the maximum effect achieved with probenecid (*n* = 3 per condition) (b). CHD and CDCHD decreased the uptake ratio into hOAT3-overexpressing cells in a concentration-dependent manner, whereas CHD exhibited a stronger effect (*n* = 3 per condition) (c). CHD and CDCHD showed an inhibitory effect on hOAT3, whereas the effect of CHD was more pronounced. The effect at 10 μM of CHD was around 75% (*n* = 3 per condition) (d). CHD and CDCHD showed a slight increase in the uptake ratio in hOAT4 cells. No inhibitory effect was observed for CHD and CDCHD (*n* = 3 per condition) (e). CHD and CDCHD showed an inverse inhibitory effect on hOAT4 in a concentration-dependent manner (*n* = 3 per condition) (f). CHD and CDCHD decreased the efflux ratio out of hMRP2-overexpressing cells in a concentration-dependent manner (*n* = 3 per condition) (g). CHD and CDCHD showed a slight inhibitory effect on hMRP2 in a concentration-dependent manner (*n* = 3 per condition) (h). Statistical testing was performed using an ordinary one-way ANOVA. ns, not significant; *: *p* < 0.05; ** : *p* < 0.01; ***: *p* < 0.001; ****: *p* < 0.0001.

Next, we looked at the effects on selected organic anion transporters. For hOAT1, a significantly decreased uptake was observed for CHD at 10 μM, whereas no significant effects on uptake were seen for CHD at 1 μM. Moreover, no effects for both tested concentrations of CDCHD were determined (Figures 4a, and S3a). The inhibitory effect on hOAT1 of CHD was concentration-dependent. No inhibitory effect was seen for CDCHD, independent of the concentration (Figure 4b). For hOAT3, a concentration-dependent uptake was observed for CHD as well as for CDCHD (Figures 4c, and S3b). Despite the observed concentration-dependent effects on hOAT3 for CHD and CDCHD, inhibitory effects at 10 μM were more pronounced for CHD (Figure 4d). For hOAT4, no decreased uptake was observed for CHD and CDCHD. Rather, an increased uptake was detected in a concentration-dependent manner, albeit not being significant (Figures 4e, and S3c). Both CDCHD and CHD showed an enhanced uptake at a concentration of 10 μM of up to 40 % (Figure 4f). No significant difference in the efflux ratio was observed for CHD and CDCHD on hMRP2 (Figure 4g). Moreover, only slight, concentration-dependent effects were detected for CHD and CDCHD (Figures 4h, and S3d). Thus, the inhibitory effects by CHD and CDCHD on hMRP2 were only marginal.

In summary, CHD exhibited concentration-dependent inhibitory effects on the murine mOat1 and mOat3 transporters and their human homologues, hOAT1 and hOAT3, located on the basolateral side. By contrast, no effect or only marginal effects of CDCHD were seen on those transporters; for mOat1, the inhibitory effect of CDCHD was only observed at 10 μM. Additionally, CHD exhibited similar effects compared to mOat1 and mOat3 on the murine mOat2 transporter, located on the apical side. Finally, no inhibitory but rather enhancing effects were observed for CHD and CDCHD on hOAT4, whereas only minor inhibition was seen on hMRP2.

### CHD and CDCHD Do Not Inhibit Organic Cation Transporters

**Figure 5:**
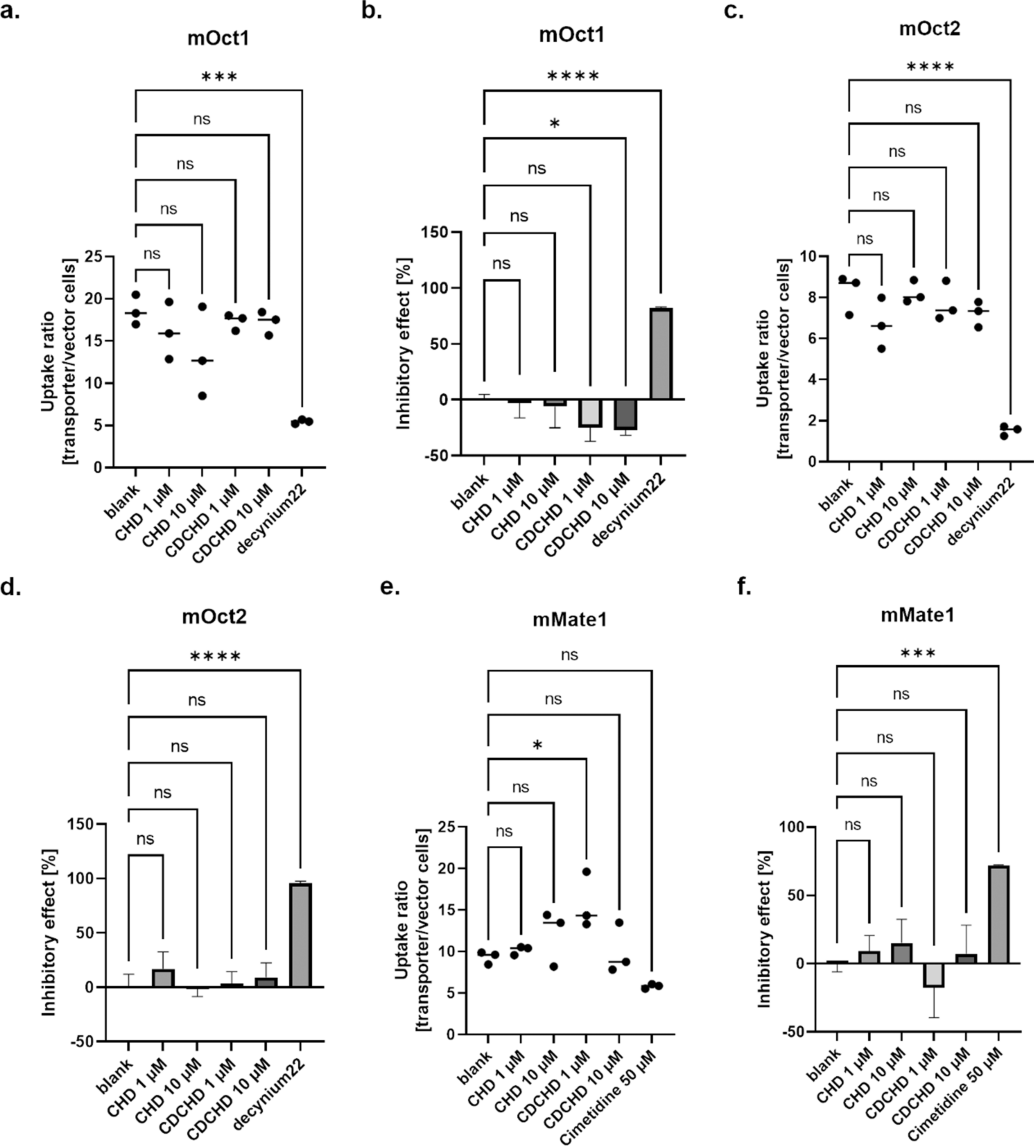
Interactions of CHD and CDCHD with murine organic cation transporters. The uptake (a, c) as well as the efflux (e) ratio and the inhibitory effect (b, d, f) were determined for mOct1 (a, b), mOct2 (c, d), and mMate1 (e, f). The uptake/efflux ratio of substrate as a function of amount per transporter/amount per vector cells was calculated for each condition (a, c, e): untreated, addition of CHD at 1 and 10 μM, addition of CDCHD at 1 and 10 μM, and addition of inhibitor. The inhibitory effect as a net uptake compared to total net uptake of blank cells in percent was calculated for each condition (b, d, f): untreated, addition of CHD at 1 and 10 μM, addition of CDCHD at 1 and 10 μM, and addition of inhibitor. A decreased uptake was seen on for CHD on the uptake1 ratio, whereas no effect was observed for CDCHD in mOct1-overexpressing cells (*n* = 3 per condition) (a). No inhibitory effect of CHD was observed, but a slight inverse inhibitory effect of CDCHD was seen, albeit not concentration-dependent. (*n* = 3 per condition) (b). CHD and CDCHD decreased the uptake ratio into mOct2-overexpressing cells only marginally (*n* = 3 per condition) (c). No inhibitory effect of CHD and CDCHD was observed on mOct2 (*n* = 3 per condition) (d). CHD and CDCHD did not show major effects on the efflux ratio of mMate1 (*n* = 3 per condition) (e). CHD showed a weak inhibitory effect on mMate1, whereas CDCHD did not have major effects on mMate1 (*n* = 3 per condition) (f). Statistical testing was performed using an ordinary one-way ANOVA. ns, not significant; *: *p* < 0.05; ***: *p* < 0.001; ****: *p* < 0.0001.

Next, we determined the potential effects on murine organic cation transporters, mOct1 and mOct2, in cell lines stably overexpressing these transporters. We assessed the uptake ratio as well as the potential inhibitory effects of CHD and CDCHD. Similar to the setup with organic anion transporters, again, the uptake of a transporter-specific probe substrate was determined in the presence of CHD and CDCHD to assess the potential inhibitory properties. As a comparator, a specific known inhibitor was used, i.e., decynium22 or cimetidine.^52^ For mOct1, a slightly decreased uptake ratio was observed for CHD, which was mainly a result of higher uptake into vector cells compared to that of untreated control cells. When considering only transporter-transfected cells, CHD did not have an effect on the substrate uptake. By contrast, the uptake in the presence of CDCHD increased. Surprisingly, this was not concentration-dependent (Figures 5a, and S4a). Neither CHD nor CDCHD had an inhibitory effect on the uptake. A similar magnitude of enhanced uptake was observed for CDCHD at both tested concentrations, whereas the effects of CHD were only marginal (Figure 5b). Moreover, no effects on uptake were seen for mOct2, for neither CHD nor CDCHD (Figures 5c, and S4b). Thus, no effect on inhibition was seen either (Figure 5d). Additionally, no major effects on uptake on mMate1 were observed for CHD and CDCHD (Figure S4c). Although CDCHD at 1 μM exhibited a slightly higher uptake ratio compared to blank control, this might be attributed to a slightly lower uptake of substrate into vector cells in the presence of CDCHD at 1 μM (Figures 5e, and S4c). Only slight inhibitory effects were observed for CHD in a concentration-dependent manner, whereas CDCHD did not show any inhibitory effects on mMate1 (Figure 5f).

In conclusion, no inhibitory effects were observed for CHD and CDCHD on the tested organic cation transporters, as well as on mMate1. By contrast, enhancing effects were detected for CDCHD for mOct1.

### CHD and CDCHD Do Not Exhibit Major Inhibitory Effects on Efflux Transporters

**Figure 6:**
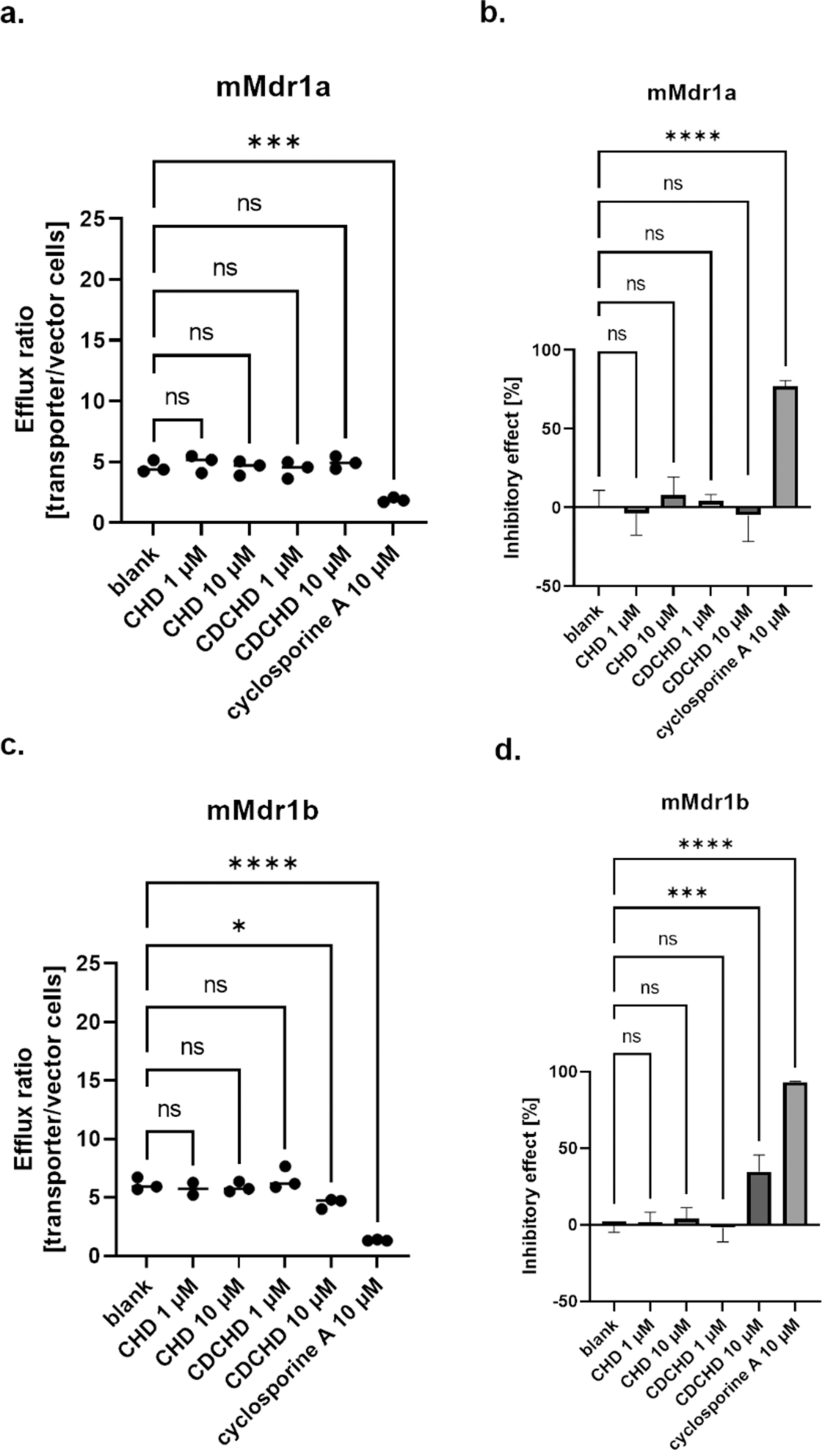
Interaction with CHD and CDCHD on mMdr efflux transporters. The efflux ratio and the inhibitory effect were determined for mMdr1a (a, b) and mMdr1b (c, d). The efflux ratio of substrate as a function of amount per transporter/amount per vector cells was calculated for each condition (a, c): untreated, addition of CHD at 1 and 10 μM, addition of CDCHD at 1 and 10 μM, and addition of inhibitor. The inhibitory effect as a net uptake compared to the total net uptake of blank cells in percent was calculated for each condition (b, d): untreated, addition of CHD at 1 and 10 μM, addition of CDCHD at 1 and 10 μM and addition of inhibitor. No effect on efflux was observed for CHD and CDCHD on mMdr1a-overexpressing cells (*n* = 3 per condition) (a). No inhibitory effect was seen for CHD or CDCHD, either (*n* = 3 per condition) (b). CHD did not decrease the efflux ratio, whereas CDCHD did at 10 μM on mMdr1b-overexpressing cells (*n* = 3 per condition) (c). CDCHD exhibited an inhibitory effect at 10 μM on mMdr1b, whereas no effect was seen for CHD (*n* = 3 per condition) (d). Statistical testing was performed using an ordinary one-way ANOVA. ns, not significant; *: *p* < 0.05; ***: *p* < 0.001; ****: *p* < 0.0001.

In a next step, we looked at two murine efflux transporters, namely, mMdr1a and mMdr1b. Both are located on the apical side and responsible for the secretion of compounds into the lumen.^35,43^ Efflux was determined in the presence of a transporter-specific substrate and an inhibitor as a comparator, i.e., cyclosporine A for both transporters. For mMdr1a, no effect was observed for CHD and CDCHD on the efflux ratio (Figure 6a). Furthermore, only small inhibitory effects for CHD at 10 μM were detected on mMdr1a (Figures 6b, and S5a). Then, we looked at mMdr1b. Here, an effect on the efflux ratio was seen for only CDCHD at 10 μM (Figure 6c). This also translated in a stronger inhibitory effect at 10 μM for CDCHD at 10 μM in the range of around 30% (Figures 6d, and S5b). However, the inhibitory effect was not as strong as seen for the comparator cyclosporine A.

In summary, CDCHD at a high concentration (10 μM) exhibited an inhibitory effect on mMdr1b, whereas no effect was observed for CHD. By contrast, no or only minor inhibition was seen on mMrd1a.

### CHD is a Substrate of mOat3 and mOct1, Whereas CDCHD is Not

As CHD and CDCHD exhibited distinct inhibitory profiles toward the tested transporters, we selected mOat1, mOat3, and mOct1 to study if CHD and CDCHD were also substrates. This is important to gain a better understanding of the underlying mechanisms of secretion into urine. Therefore, we assessed the amount of compound taken up by the vector and transporter cells at three different concentrations (1, 10, and 100 μM for mOat1 and mOct1; 0.1, 1, and 10 μM for mOat3) and at two different incubation periods (1 and 5 min for mOat1 and mOct1; 5 and 10 min for mOat3). Additionally, we added known substrate inhibitors at the CHD or CDCHD concentration of 10 μM for two different incubation periods to prove that uptake depends on the selected transporter.^42,52^

**Figure 7:**
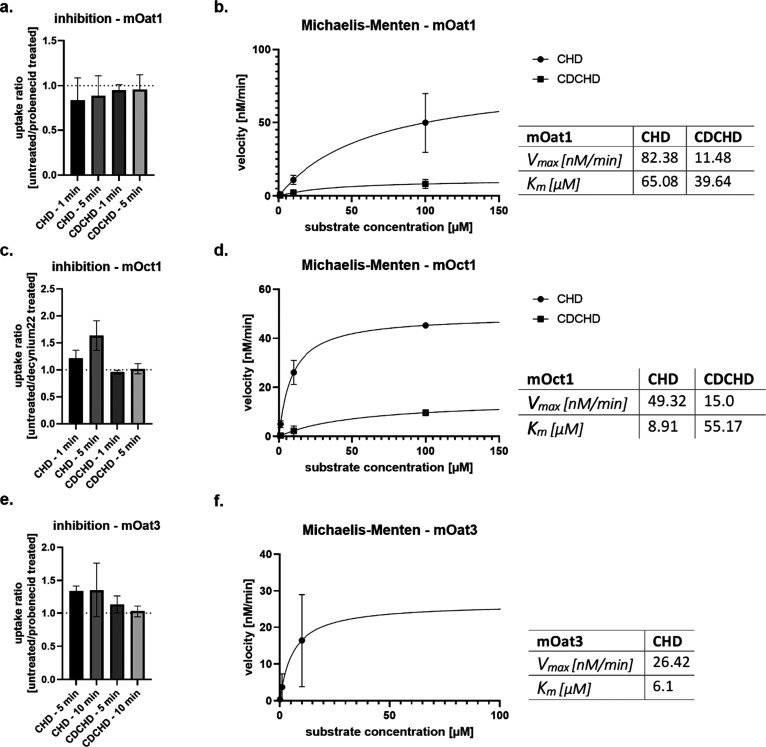
Assessment of CHD and CDCHD as substrates of mOat1, mOct1, and mOat3. The uptake ratio (a, c, e) as well Michaelis–Menten kinetics (b, d, f) were assessed for CHD and CDCHD in mOat1- (a, b), mOct1- (c, d,), and mOat3-overexpressing (e, f) cells. The uptake ratio for CHD and CDCHD was determined for each incubation time point as a function of untreated cells in relation to probenecid (a, e) or decynium22 (c) treated cells. Michaelis–Menten kinetics were determined for CHD and CDCHD using three concentrations per substrate. *K*_m_ and *V*_max_ were calculated using the Michaelis–Menten algorithm of GraphPad Prism software. Uptake ratio of CHD was decreased, whereas CDCHD was not. This suggested no inhibition of transport (*n* = 3 per condition) (a). Michaelis–Menten kinetics were determined for CHD and CDCHD. Low affinities to mOat1 were observed underlining that both are not substrates of mOat1 (*n* = 3 per concentration) (b). CHD showed an increased concentration-dependent uptake ratio, whereas CDCHD was not affected. This suggested mOct1-specific transport of CHD (*n* = 3 per condition) (c). Michaelis–Menten kinetics were determined for CHD and CDCHD. Low affinity to mOct1 was observed for CDCHD, whereas CHD showed high affinity (*n* = 3 per concentration) (d). CHD showed an increased uptake ratio, whereas CDCHD was not affected. This suggested mOat3-specific transport of CHD (*n* = 3 per condition) (e). Michaelis–Menten kinetics were determined for CHD. High affinity to mOat3 was observed for CHD (*n* = 3 per concentration) (f).

For CDCHD, uptake was similar for the vector- and mOat1-expressing cells at all tested concentrations. For 1 and 10 μM, similar amounts were detected intracellularly, whereas this only slightly increased at 100 μM (Figure S6a). No inhibitory effect of probenecid was observed for CDCHD at both incubation time points (Figure S6b,c). When looking at the uptake ratio between cells without and with probenecid as the inhibitor of mOat1, no inhibitory effect was seen, either (Figure 7a). For CHD, the uptake increased from 1 to 5 min incubation period, as higher concentrations were detected intracellularly for both the vector- and mOat1-expressing cells at 5 min (Figure S6a). Moreover, the intracellular concentrations of CHD increased with the increasing concentrations of CHD. However, this was observed for both the vector- and mOat1-expressing, cells (Figure S6a), pointing to a passive membrane permeation of the drug. No inhibitory effect of probenecid on the CHD cellular uptake was observed at 1 and 5 min time points (Figure S6b,c). Rather, a slight increase in uptake was seen for CHD upon the addition of probenecid, which is also reflected in the uptake ratio, which was, for both time points, slightly lesser than 1 (Figure 7a). We calculated *K*_m_ and *V*_max_ for CHD and CDCHD as we assumed Michaelis–Menten kinetics for mOat1. Here, we observed a high *K*_m_ for CHD and CDCHD, suggesting a low substrate affinity for mOat1 (Figure 7b).

Similar to mOat1, also for mOct1, the CDCHD uptake was similar, irrespective of the incubation time or cell type, i.e., vector- or mOct1-expressing cells (Figure S7a). A significant inhibitory effect of decynium22 on CDCHD uptake into mOct1-expressing cells was not observed either at both incubation time points (Figure S7b,c). This was also reflected in the uptake ratio, which was 1 for CDCHD (Figure 7c). For CHD, a concentration-dependent uptake was seen for both cell types (vector- and mOct1-expressing cells; Figure S7a). Moreover, a decreased uptake of CHD was detected upon the addition of decynium22 at both tested incubation times (Figure S6b,c). The uptake ratio for CHD increased at longer incubation periods (i.e., 5 min compared to 1 min) upon the addition of decynium22, resulting in around 50% less uptake in the presence of a specific inhibitor (Figure 7c). Next, under the assumption of Michaelis–Menten kinetics, we determined *K*_m_ and *V*_max_ for CHD and CDCHD. Transport for CHD was saturated at 100 μM, as with the calculated *V*_max_ around 49 nM/min. Moreover, CHD showed higher affinity for mOct1 compared to CDCHD (*K*_m_(CHD) = 8.91 μM vs *K*_m_(CDCHD) = 55.2 μM), which was in line with the data upon the inhibition of CHD uptake with decynium22 (Figure 7d).

Next, we determined the cellular uptake of CHD and CDCHD in the vector- and mOat3-expressing cells. Whereas no major increase in the intracellular concentration of CDCHD upon ascending concentrations was observed, intracellular concentration of CHD augmented with increasing concentrations (Figure S7a). Furthermore, the increase of the incubation period also slightly resulted in higher intracellular concentrations of CHD, whereas that was not the case for CDCHD (Figure S8a). A slight inhibition of uptake was seen for CHD upon the addition of probenecid (Figure S8b,c). This was not observed for CDCHD (Figure S8b,c). The uptake ratio showed a clear inhibition of uptake for CHD but not for CDCHD. However, for CHD, the inhibition was not incubation time-dependent (Figure 7e). Next, we determined *K*_m_ and *V*_max_ under the assumption of Michaelis–Menten kinetics for mOat3. Whereas *K*_m_ and *V*_max_ were not calculated for CDCHD, as intracellular concentrations were too low for all three substrate concentrations used, CHD showed high affinity with a *K*_m_ value of around 6.1 μM (Figure 7f).

In summary, CHD is a substrate of mOct1 as well as mOat3, whereas CDCHD is not a substrate of these two transporters. With regard to mOat1, it was shown that both CHD and CDCHD were not substrates of this transporter.

## Discussion

In this study, we elucidated the potential interactions of CHD and CDCHD with several renal drug transporters. We selected organic anion and cation transporters as well as efflux transporters for both species, with a focus on murine transporters, but also included human homologues. Thereby, we assessed the inhibitory potential on these transporters and investigated for mOat1, mOat3, and mOct1 if CHD and CDCHD are substrates. For all the three generations of tetracyclines, no involvement of renal drug transporters in clearance is published to date, apart from omadacycline which is transported by p-gp and has a relatively high affinity for p-gp (*K*_m_ ∼ 82 μM)^53^. For several tetracyclines, such as tigecycline or omadacycline, it was reported that renal impairment did not result in changes in PK behavior.^54−55,56^

In general, CHD and CDCHD showed a high residual penetration into vector cells, irrespective of inhibition. This might be expected as both compounds do have a high volume of distribution in vivo, suggesting good penetration into tissue and rapid distribution from plasma.^29^ A similar behavior when conducting organic anion transporter assays was observed for omadacycline with a similarly high volume of distribution and good penetration into the tissue.^53^ Thus, for the assessment of substrate specificity, it was important to consider transporter-overexpressing cells with and without inhibitors. This enabled to detect small differences in transport, albeit high residual penetration into the in vitro cell system, irrespective of the overexpressed transporter.

CHD and CDCHD did not exhibit inhibitory effects on mMdr1a, i.e., the homologue of p-gp.^50^ Moreover, only CDCHD had an inhibitory effect at 10 μM on mMdr1b, whereas CHD did not have an effect at all. Studies investigating the drug–drug interaction potential of different compounds from the class of tetracyclines reveal them as substrates rather than inhibitors of p-gp with an overall low drug-drug interaction potential. One study demonstrated that the simultaneous administration of cyclosporine A, an inhibitor of MDR1/Mdr1, with different tetracyclines resulted in an increase of AUC and a reduced clearance, even after intravenous administration of the respective tetracycline, suggesting the involvement of these transporters in the clearance of tetracyclines.^57^ The simultaneous administration of tigecycline and digoxin, another p-gp (MDR) substrate^58^, in healthy men showed no effect on the PK of digoxin, whereas the effect on tigecycline was not ruled out.^59^ Similarly, the p-gp (MDR) inhibitor verapamil increased the AUC of perorally administered omadacycline, although that was not clinically relevant.^60^ However, these results were in line with the in vitro results obtained by Flarakos and colleagues.^53^ Our study has the limitation that we only assessed if CHD and CDCHD were inhibitors of mMdr1a and mMdr1b. However, the results suggest that CHD and CDCHD, despite being atypical tetracyclines, might exhibit a similar behavior to ‘classical’ tetracyclines.

CHD and CDCHD had different inhibitory signatures toward organic anion and cation transporters: Whereas CHD exhibited significant inhibitory properties toward transporters located at the basolateral side (mOat1, mOat3, hOAT1, and hOAT3), CDCHD inhibited those transporters only marginally (mOat1, hOAT3) or not inhibited (mOat3 and hOAT1). Interestingly, CHD and CDCHD also differed in their substrate specificity: whereas CHD was a substrate of mOat3, CDCHD was a substrate of neither mOat1 nor mOat3. CHD had a high affinity to mOat3 (*K*_m_ ∼ 6 μM) and already demonstrated up to 50% transport inhibition at 10 μM according to our preliminary Michaelis–Menten kinetic analysis with a limited number of data points. These concentrations are reached in vivo in urine^29^. CHD inhibited mOat1 and mOat3 and the corresponding human transporters hOAT1 and hOAT3 in a concentration-dependent manner. Moreover, the inhibition of hOAT3 by CHD appeared to be stronger than that of mOat3. One limitation of the study is that only inhibition of human transporters was assessed, but not if equally to mOat3 CHD is a substrate of hOAT3. Also, omadacycline which inherits the same amide-functionality as CDCHD (Figure 1) is neither a substrate nor an inhibitor of organic anion transporters.^53^ One might hypothesize that the lack of amide-functionality in CHD might be a reason for the different substrate specificity for mOat3. We hypothesize that the better and higher affinity of CHD to Oats was a result of the carboxyl group, which is lacking in CDCHD and omadacycline, since, for example, PAH similar to the OAT1 probe substrate also possesses a comparable carboxyl group and a similar negative charge of the molecule. To get a better understanding of the structure–substrate relationships, further studies with CHD- and CDCHD-analogues could be envisaged in case they show similar promising PK and pharmacodynamic results. Furthermore, CHD inhibited reabsorption into the proximal tubule cell via mOat2 on the apical side in a concentration-dependent manner. CDCHD did only inhibit that transporter at 10 μM. For the human organic anion transporters located at the apical side, CDCHD and CHD showed either similar inhibition as for hMRP2 or an inverse inhibitory effect as for hOAT4. This inverse inhibitory effect was also observed for mOct1, whereas no inhibitory effect was seen on mOct2 and mMate1 for CHD and CDCHD. However, as the assays were not designed to assess the induction of transporters, one might speculate that CHD and CDCHD caused enhanced substrate transport and, thereby, were responsible for the observed inverse inhibitory effect. It has been described in early in vitro models in the late 1970s that nephrotoxic substances can cause an enhanced substrate transport.^61,62^ Despite the observed effect on mOct1 by both atypical tetracyclines, only CHD was a substrate with moderate affinity (*K*_m_ ∼ 8.9 μM) according to our preliminary Michaelis–Menten kinetic analysis with a limited number of data points. It is known for ciprofloxacin, a fluoroquinolone, and ceftriaxone, a cephalosporin, that they can exhibit nephrotoxic side effects. Both antibacterials are transported via either hOAT1 or hOAT3, thus, via basolateral organic anion transporters. It is assumed that nephrotoxicity of these two antibacterials might be a result of accumulation in the renal proximal tubule cells due to inhibition of another efflux transporter on the apical side.^63^ CHD was also transported via mOat3 and inhibited mOat3 as well. Moreover, CHD inhibited hOAT3 and at the same time also hMRP2, an efflux transporter on the apical side, whereas CDCHD inhibited only hMPR2. We did not assess if CHD or CDCHD were the substrates of hMRP2, but transport via hOAT1/3 or mOat1/3 and inhibition of secretion into the lumen on the apical side via hMRP2 could have the risk of accumulation and, consequently, nephrotoxicity due to crystallization in the tubules, similar to ciprofloxacin or ceftriaxone^63^. The transporters mOat3 and mOct1 are both located in the proximal tubule cells. Thus, this suggested the transport of CHD via mOat3 and mOct1 and inhibition of mOat3 at high concentrations. In our previous study,^29^ we observed that those concentrations, needed to inhibit mOat3 as seen here, were already reached in plasma and urine after 15 mg/kg IV in vivo. Moreover, we showed in our previous study that the urine concentrations of CHD decreased rapidly after 8 h post-administration, whereas CDCHD concentrations remained at a high level. Additionally, CHD had 10-fold higher urine concentrations up to 8 h post administration in urine compared to CDCHD.^29^ Consequently, it is conceivable that the cause for the rapid decrease in the urine concentrations of CHD compared to CDCHD is a result of active transport into renal tubule cells, which was not inhibited at lower concentrations anymore. By contrast, CDCHD was not actively transported but rather reached the renal tubule cells via passive diffusion due to its lipophilicity and appeared in urine as a result of glomerular filtration resulting in more sustained urine levels. From the phase II clinical trial conducted in the 1970s, it is known that CHD reached high concentrations in urine (range 11–14 μg/mL), whereas plasma concentrations were much lower (range 1–3 μg/mL).^24^ This observation was similar to the PK behavior seen in mice.^29^ Although irreversible toxicity of CHD and CDCHD has not been observed during the histopathological analysis of kidneys and livers from PK samples in our previous study,^29^ it is important to consider that CHD clinical development had been stopped despite encouraging phase II trial results.^24^ Considering the potential for nephrotoxicity for CHD and CDCHD because of the inhibition of transporters on the apical side as well as the end of development of CHD and the yellow discoloration observed in kidneys in our previous study,^29^ future studies will be imperative to specifically assess the nephrotoxic potential at different doses of both CHD and CDCHD.

In conclusion, this study was designed to get a more in-depth understanding of the PK behavior observed in our previous study elucidating preliminary PK/PD relationships of CHD and CDCHD.^29^ We demonstrated that CHD was actively transported via mOat3 and mOct1, whereas CDCHD was not a substrate of the investigated basolateral transporters. Additionally, CHD was a strong inhibitor of several basolateral transporters, such as mOat1 and mOat3, as well as their human homologues hOAT1 and hOAT3. By contrast, CDCHD showed a different inhibition profile of basolateral transporters compared to CHD. Furthermore, CHD and CDCHD inhibited several apical transporters and caused enhanced substrate transport.

In summary, these findings suggested that CHD might cause an autoinhibition on mOat3, leading to initially higher plasma concentrations, as observed in vivo.^29^ This inhibition of active transport into the tubular epithelial cells at high concentrations in conjunction with a potential inhibition of apical transporters responsible for tubular secretion into urine might lead to accumulation. This can result in subsequent nephrotoxicity, which requires further investigation. Additionally, the inhibition of apical transporters by CDCHD preventing its active secretion into urine could lead to the observed 10-fold lower concentrations in urine compared to CHD but equal to the accumulation of parent compound in the tubule cells. Accumulation of both, CHD and CDCHD, was suspected previously because of the yellow discoloration of kidneys.^29^

Finally, this investigation of the transporter substrate- and inhibitor-specific properties of CHD and CDCHD helps explaining the distinct PK profiles observed for CHD compared to CDCHD, in particular with respect to plasma and urine concentrations.^29^ In particular, the observation of an accumulation potential of CHD and CDCHD in the proximal tubule cells warrants the assessment of the nephrotoxic potential at different doses of CHD and CDCHD before further preclinical development.

## Materials and Methods

### Chemicals

1-Methyl-4-phenylpyridinium iodide (MPP) was used as a substrate for mOct1 and mOct2. [^3^H]-MPP (Methyl-4-phenylpyridinium iodide, 1-[methyl-^3^H]) was purchased from American Radiolabeled Chemicals. 1,1′-Diethyl-2,2′-cyanine iodide (Decynium 22) was used as an inhibitor for mOct1 and mOct2. Metformin (1,1-dimethylbiguanide hydrochloride) was purchased from Sigma-Aldrich and was used as a probe substrate for mMate1. Metformin (biguanido-^14^C) hydrochloride was purchased from American Radiolabeled Chemicals. Cimetidine was purchased from Sigma-Aldrich and was used as a probe inhibitor for mMate1. p-Aminohippuric acid (PAH) was purchased from Sigma-Aldrich and was used as a substrate for mOat1 and hOAT1. Estrone 3-sulfate (ES) was used as a substrate for mOat3, hOAT3, and hOAT4. ^3^H-Estrone sulfate, ammonium salt, and [6,7-^3^H(N)] were purchased from American Radiolabeled Chemicals. Sulfobromophthalein (BSP) was used as an inhibitor for hOAT4. 5(6)-Carboxy-2′,7′-dichlorofluorescein diacetate (CDCF-DA) was purchased from Sigma-Aldrich and was used as a fluorescent substrate for hMRP2. 5(6)-Carboxy-2′,7′-dichlorofluorescein (CDCF) was purchased from Sigma-Aldrich and was used as a fluorescent substrate for the calibration curve. Benzbromarone was used as a probe inhibitor for hMRP2. Probenecid (*p*-(Dipropylsulfamoyl) benzoic acid) was used as an inhibitor for mOat1 as well as for mOat3, hOAT1, and hOAT3. Rhodamin 123 (fluorescent dye) was used as a probe substrate for mMdr1a and mMdr1b. Cyclosporine A was used as a probe inhibitor for mMdr1a and mMdr1b.

### Preparation, Isolation, and Formulation of CHD and CDCHD

CHD and CDCHD were prepared and isolated as described previously.^29^ Additionally, CHD and CDCHD were formulated as described previously to allow good dissolution in cell medium for the in vitro experiments.^29,64^

### Cells

Well-characterized mOct1-, mOct2-, mOat1-, mOat2-, mOat3-, mMate1-, hOAT1-, hOAT3-, hOAT4-, hMRP2-, mMdr1a- and mMdr1b-transfected HEK293 cells and the respective vector-transfected control HEK293 cells were used.^42,51,52^ All HEK293 cells (transporter-transfected and vector-transfected) were cultured in DMEM (Sigma, high glucose, 10% FBS, 1% penicillin (10,000 Units/mL)/streptomycin (10 mg/mL)) at 37°C under 5% CO_2_ in humidified atmosphere. The cells were routinely checked for the absence of mycoplasma infection. For the preparation of HEK293 cell monolayers, 24-well plates were pretreated with a poly-D-lysine hydrobromide solution (0.1 mg/mL). Each well was coated with 0.5 mL of poly-D-lysine solution and incubated for at least 15 min. After complete removal of the solution, the plates were dried for 30 min. Cells (vector-transfected and transporter-transfected) treated with trypsin were immediately seeded into 24-well plates and cultured for 3 days before being used for transport experiments.

### Uptake Transport Assay

For the uptake assay, the growth medium was aspirated, and each well was rinsed three times with 0.5 mL of incubation buffer (HBSS supplemented with 20 mM HEPES, pH 7.4) and incubated for 20 min at 37 °C. Afterward, buffers were removed, and 200 μL of the incubation buffer containing a radiolabeled substrate and the nonlabeled CHD and CDCHD or reference inhibitor (Table S1), respectively, was added to each well and incubated at 37 °C for designated time intervals. After incubation, uptake was terminated by aspirating the reaction mixture and washing the cells three times with 0.4 mL of ice-cold incubation buffer. Cells were solubilized with 0.6 mL of 1N NaOH overnight and transferred into scintillation vials. ^3^H and ^14^C contents were measured after the addition of 2.5 mL of scintillator in a liquid scintillation counter.

The cellular protein amount was determined in parallel for each cell line (vector- and transporter-transfected) from six wells in a different 24-well reference plate using the Bradford method. For the two-point inhibition assays, each, CHD and CDCHD, was added in two single concentrations of 1 and 10 μM to characterize their inhibitory potential. Incubation was terminated after 1 min. All experiments were conducted in triplicate for every transporter.

### Two-Point Inhibition: Inhibition of mOct1 and mOct2-Mediated Uptake of MPP

The MPP incubation buffer contained ^3^H-MPP and nonlabeled MPP in a final concentration of 10 μM. Inhibition of mOct1 and mOct2-mediated MPP uptake by 50 μM decynium22 was performed as a control experiment.

### Two-Point Inhibition: Inhibition of mOat1-Mediated Uptake of PAH

The PAH incubation buffer contained ^3^H-PAH and nonlabeled PAH in a final concentration of 10 μM. Inhibition of mOat1-mediated PAH uptake by 100 μM probenecid was performed as a control experiment.

### Two-Point Inhibition: Inhibition of mOat2-Mediated cGMP Uptake

The cGMP incubation buffer contained ^3^H-cGMP and nonlabeled cGMP at a final concentration of 10 μM. Inhibition of mOat2-mediated cGMP uptake by 10 μM indomethacin was performed as the control experiment. Here, after 5 min, the incubation was terminated. The experiment was performed in sextuplicate.

### Two-Point Inhibition: Inhibition of mOat3-Mediated Uptake of ES

The ES incubation buffer contained ^3^H-ES and nonlabeled ES in a final concentration of 1 μM. Inhibition of mOat3-mediated ES uptake by 100 μM probenecid was performed as a control experiment.

### Two-Point Inhibition: Inhibition of mMate1-Mediated Uptake of Metformin

The metformin incubation buffer contained ^14^C-metformin and nonlabeled metformin in a final concentration of 20 μM. Inhibition of mMate1-mediated metformin uptake by 50 μM cimetidine was performed as a control experiment.

### Two-Point Inhibition: Inhibition of hOAT1-Mediated Uptake of PAH

The PAH incubation buffer contained ^3^H-PAH and nonlabeled PAH in a final concentration of 10 μM. Inhibition of mOat1-mediated PAH uptake by 100 μM probenecid was performed as a control experiment.

### Two-Point Inhibition: Inhibition of hOAT3-Mediated Uptake of ES

The ES incubation buffer contained ^3^H-ES and nonlabeled ES in a final concentration of 1 μM. Inhibition of mOat3-mediated ES uptake by 100 μM probenecid was performed as a control experiment.

### Two-Point Inhibition: Inhibition of hOAT4-Mediated Uptake of ES

The ES incubation buffer contained ^3^H-ES and nonlabeled ES in a final concentration of 1 μM. Inhibition of hOAT4-mediated ES uptake by 10 μM BSP was performed as a control experiment.

### Data Analysis of Two-Point Inhibition of Uptake Assays

The absolute amount (picomoles) of the substrate uptake was calculated within the given time and related to the determined protein values. Initially, the specific activity was calculated according to the labeling (μCi/mmol) and the concentration of the substrate. Additionally, the radioactivity (dpm) of an aliquot of each substrate solution was determined. From this, the specific radioactivity, the so-called “*standard*”, was calculated according to the following formula:
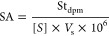
1SA, specific radioactivity of the standard (dpm/pmol); St_dpm_, mean radioactivity of standards (dpm); [*S*], substrate concentration (μmol/L); and *V*_s_, volume of the aliquot substrate solution (L).

The specific radioactivity from each condition was used to determine the uptake values *U* of every sample, which was calculated according to the following formula:
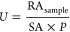
2*U*, uptake (pmol/mg protein); SA, specific radioactivity (dpm/pmol); RA_sample_, radioactivity of one well of the 24-well plate (dpm); and *P*, protein amount (mean of six wells from the 24-well reference plate) (mg protein).

Initial uptake rate *v* for each well was calculated according to the following formula:
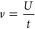
3*v*, uptake rate (pmol/mg protein/min); *U*, uptake (pmol/mg protein); and *t*, incubation interval (min).

Transporter-mediated uptake rate (net uptake) was obtained by subtracting the uptake rate in vector-transfected HEK cells from the uptake rate in transporter-HEK293, as described below:
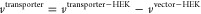
4*v*^transporter^, initial uptake rate (transporter-mediated) (pmol/mg protein/min); *v*, initial uptake rate (pmol/mg protein/min).

The inhibitory effect (*I*(%)) was calculated according to the formula:
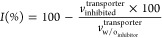
5*v*_inhibited_^transporter^, transporter-mediated uptake (= net uptake) with the inhibitor (pmol/mg protein/min); *v*_w/o_inhibitor__^transporter^, transporter-mediated uptake (= net uptake) without the inhibitor (pmol/mg protein/min); and *I* (%), inhibitory effect.

The probe substrate uptake ratio between transporter-transfected and vector-transfected HEK cells was calculated to prove the functionality of the test system. The following equation was applied:
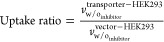
6*v*_w/o_inhibitor__^transporter–HEK293^, initial uptake rate in transporter HEK 293 cells without the inhibitor (control) (pmol/mg protein/min); and *v*_w/o_inhibitor__^vector–HEK293^, initial uptake rate in vector HEK 293 cells without the inhibitor (control) (pmol/mg protein/min).

### Three-Point Uptake in mOat1

The incubation buffer contained CHD and CDCHD in a final concentration of 1, 10, and 100 μM. After 1 and 5 min, the incubation was terminated. In addition, the probe inhibitor probenecid (100 μM) was added in one concentration to 10 μM CHD and CDCHD, respectively, and after 1 and 5 min, the incubation was stopped. Inhibition of mOat1-mediated ^3^H-PAH (10 μM) uptake by 100 μM probenecid was performed as a control experiment. After 1 min, the incubation was terminated. All experiments were conducted in triplicate.

### Three-Point Uptake in mOct1

The incubation buffer contained CHD and CDCHD in a final concentration of 1, 10, and 100 μM. After 1 and 5 min, the incubation was terminated. In addition, the probe inhibitor decynium22 (50 μM) was added in one concentration to 10 μM CHD and CDCHD, respectively, and after 1 and 5 min, the incubation was stopped. Inhibition of mOct1-mediated ^3^H-MPP (10 μM) uptake by 50 μM decynium22 was performed as a control experiment. After 1 min, the incubation was terminated. All experiments were conducted in triplicate.

### Three-Point Uptake in mOat3

The incubation buffer contained CHD and CDCHD in a final concentration of 0.1, 1, and 10 μM. After 5 and 10 min, the incubation was terminated. In addition, the probe inhibitor probenecid (100 μM) was added in one concentration to 10 μM CHD and CDCHD, respectively, and after 5 and 10 min, the incubation was stopped. Inhibition of mOat3-mediated ^3^H-ES (1 μM) uptake by 100 μM probenecid was performed as a control experiment. After 1 min, the incubation was terminated. All experiments were conducted in triplicate.

### Efflux Transport Assay

For efflux assays, growth medium was aspirated, and each well was rinsed twice with 0.5 mL of incubation HBSS buffer (supplemented with 20 mM HEPES, pH 7.4) and then preincubated in 200 μL of incubation buffer containing 10 μM rhodamin123 and 10 μM cyclosporine A for 60 min at 37 °C for loading the cells with rhodamin123. After preloading, the incubation buffer was removed, cells were washed twice with cold HBSS incubation buffer, and for the efflux, 0.2 mL of incubation buffer was added to each well containing the nonlabeled CHD and CDCHD or reference inhibitor (Table S1) and incubated at 37 °C for designated time intervals. After incubation, the supernatant was transferred into a new 24-well plate. Samples were diluted with incubation buffer, and fluorescence was measured in a 96-well plate (Sarstedt, flat bottom) at an extinction of 490 nm and emission of 535 nm.

Cellular protein amount was determined in parallel for each cell line (vector- and transporter-transfected) from six wells in a different 24-well reference plate using the Bradford method.

### Two-Point Inhibition: Inhibition of mMdr1a- and mMdr1b-Mediated Efflux of Rhodamin123

The incubation buffer contained rhodamin123 in a final concentration of 10 μM. To characterize the inhibitory potential of CHD and CDCHD, each was added in two single concentrations of 1 and 10 μM. Inhibition of mMdr1a- and mMdr1b-mediated rhodamin123 efflux by 10 μM cyclosporine A was performed as a control experiment. The efflux was terminated after 30 min. All experiments were conducted in triplicate.

### Two-Point Inhibition: Inhibition of hMRP2-Mediated Efflux of CDCF

The incubation buffer contained CDCF-DA in a final concentration of 5 μM. Inhibition of hMRP2-mediated CDCF efflux by 100 μM benzbromarone was performed as a control experiment. Here, the efflux was terminated after 10 min.

### Data Analysis of Efflux Assays

The absolute amount (picomoles) of the substrate efflux was calculated within the given time and related to the determined protein values. For quantification of fluorescence values, a calibration curve of rhodamin123 was used. The fluorescence from each condition was used to determine the efflux value of every sample, which was calculated according to the following formula:
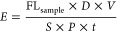
7*E*, efflux (pmol/mg protein/min); FL_sample_, fluorescence of one well of the 24 well plate; *D*, dilution; *V*, volume of the incubation buffer for efflux (L); *S*, slope of the calibration curve (1/μM × 1,000,000); *P*, protein amount (mean of six wells from the 24-well reference plate) (mg protein); and *t*, incubation interval (min).

mMdr1a- and mMdr1b-transporter-mediated efflux rate (net efflux) was obtained by subtracting the efflux rate in vector-transfected HEK293 cells from the efflux rate in mMdr1-transfected cells as described below:
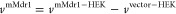
8*v*^mMdr1^, initial efflux rate (mMdr1 mediated) (pmol/mg protein/min); and *v*, initial efflux rate (pmol/mg protein/min).

The inhibitory effect *I* (%) and the efflux ratio was calculated in the same way described for the uptake assays.

### Bioanalysis of Cell Samples from Three-Point Inhibition Assays

First, a calibration curve was prepared by spiking different concentrations of CHD and CDCHD into the cell matrix. Caffeine was used as an internal standard. In addition, quality control samples (QCs) were prepared for CHD and CDCHD in a cell matrix. The following extraction procedures were used: 300 μL of acetonitrile/water (1:1) is added to the cell pellet (sample or calibration/QC sample) for 15 min at 2000 rpm on an Eppendorf MixMate vortex mixer. 10 μL of this extracted sample is used for further analysis. 10 μL of the extracted cell sample is further extracted with 25 μL of a mixture of methanol/acetonitrile (2:1) containing 12.5 ng/mL caffeine as an internal standard (on ice) for 5 min at 2000 rpm on an Eppendorf MixMate vortex mixer. Then, samples were spun down at 15,870 × *g* for 10 min. Supernatants were transferred to standard HPLC glass vials. Samples were analyzed using an Agilent 1290 Infinity II HPLC system coupled to an AB Sciex QTrap6500plus mass spectrometer. LC conditions were as follows: column: Agilent Zorbax Eclipse Plus C18, 50 × 2.1 mm, 1.8 μm; temperature: 30°C; injection volume: 5 μL per sample; flow rate: 700 μL/min. Samples were run under the following conditions. Solvents: A: 100% water +0.1% formic acid; solvent B: 100% acetonitrile +0.1% formic acid. The gradient for CDCHD was as follows: 99% A at 0 min, 99% A until 0.1 min, 99–90% A from 0.1 to 1.0 min, 90–50% A from 1.0 to 2.4 min, 50–35% A from 2.4 to 2.7 min, 35–0% A from 2.7 until 4.5 min, 0% A until 5.5 min, 0–99% from 5.5 to 5.7 min, 99% A until 6.0 min. The gradient for CHD was as follows: 99% A at 0 min, 99% A until 0.1 min, 99–90% A from 0.1 to 1.0 min, 90–55% A from 1.0 to 2.3 min, 55–40% A from 2.3 to 2.5 min, 40–0% A from 2.5 until 4.5 min, 0% A until 5.5 min, 0–99% from 5.5 to 5.7 min, 99% A until 6.0 min. Mass transitions are depicted in Table S2.

### Statistical Testing

For statistical testing of differences in uptake ratio or for the inhibitory effect, an ordinary one-way ANOVA test was performed using GraphPad Prism 9.5.1 software. For statistical testing of difference in uptake on vector- or transporter-overexpressing cells, an ordinary two-way ANOVA test was performed using GraphPad Prism 9.5.1 software. Michaelis–Menten kinetics were determined using the Michaelis–Menten algorithm of GraphPad Prism 9.5.1 software. Image resolution was adjusted by using GIMP software.

## Supporting Information Available

Uptake of substrate into mOat1-, mOat2- and mOat3-overexpressing cells upon addition of CHD and CDCHD; uptake of substrate into hOAT1-, hOAT3-, hOAT4-, and hMRP2-overexpressing cells upon the addition of CHD and CDCHD; uptake of substrate into mOct1-, mOct2-, and mMate1-overexpressing cells upon the addition of CHD and CDCHD; efflux of substrate of mMdr1a- and mMdr1b-overexpressing cells upon the addition of CHD and CDCHD; intracellular concentrations of CHD and CDCHD in mOat1-overexpressing cells; intracellular concentrations of CHD and CDCHD in mOct1-overexpressing cells; intracellular concentrations of CHD and CDCHD in mOat3-overexpressing cells; inhibitors as well as substrates including their concentrations per transporter; and mass transitions for CHD and CDCHD using caffeine as an internal standard (PDF)
