# Supplementary material for: Interaction of the Atypical Tetracyclines Chelocardin and Amidochelocardin with Renal Drug Transporters
Source: ACS Pharmacol Transl Sci. 2024 Jun 11;7(7):2093–109. doi: 10.1021/acsptsci.4c00183 (PMC11249637; doi:10.1021/acsptsci.4c00183)
Supplement: Supplementary file 1 — pt4c00183_si_001.pdf [file pt4c00183_si_001.pdf]

Supporting Information

Interaction of the atypical tetracyclines chelocardin and amidochelocardin with renal drug transporters

Katharina Rox[†, ‡], Annett Kühne[§], Jennifer Herrmann[‡,∥], Rolf Jansen[⊥], Stephan Hüttel[‡,⊥], Steffen Bernecker[⊥], Yohannes Hagos[§], Mark Brönstrup[†, ‡], Marc Stadler[‡,⊥], Thomas Hesterkamp[‡,#], Rolf Müller[‡,∥,*]
[*] Correspondence:

Prof. Dr. Rolf Müller: rolf.mueller@helmholtz-hips.de, phone: +49 681 98806 3000

Table of content

Supporting figures S-3

Figure S1. Schematic illustration of the transporter-specific probe substrate and the investigation of inhibition of transport. S-3

Figure S2. Uptake of substrate into mOat1-, mOat2- and mOat3-overexpressing cells upon addition of CHD and CDCHD. S-4

Figure S3. Uptake of substrate into hOAT1-, hOAT3-, hOAT4- and hMRP2-overexpressing cells upon addition of CHD and CDCHD. S-5

Figure S4. Uptake of substrate into mOct1-, mOct2- and mMate1-overexpressing cells upon addition of CHD and CDCHD. S-6

Figure S5. Efflux of substrate of mMdr1a- and mMdr1b-overexpressing cells upon addition of CHD and CDCHD. S-7

Figure S6. Intracellular concentrations of CHD and CDCHD in mOat1-overexpressing cells. S-8

Figure S7. Intracellular concentrations of CHD and CDCHD in mOct1-overexpressing cells. S-9

Figure S8. Intracellular concentrations of CHD and CDCHD in mOat3-overexpressing cells. S-10

Supporting tables S-11

Table S1. Inhibitors as well as substrates including their concentrations per transporter S-11

Table S2. Mass transitions for CHD and CDCHD using caffeine as internal standard S-11

Supporting figures

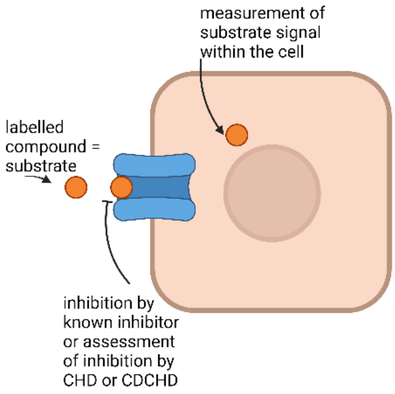

Figure S1. Schematic illustration of the transporter-specific probe substrate and the investigation of inhibition of transport.

The probe substrate (orange) is transported via a specific transporter (blue) into the cell. There, the signal of the probe substrate equivalent to the concentration is measured. In addition, a known inhibitor or CHD and CDCHD are added. In case, transport is inhibited, less probe substrate is found in the cell, i.e. less signal of the probe substrate is obtained. Created with biorender.com

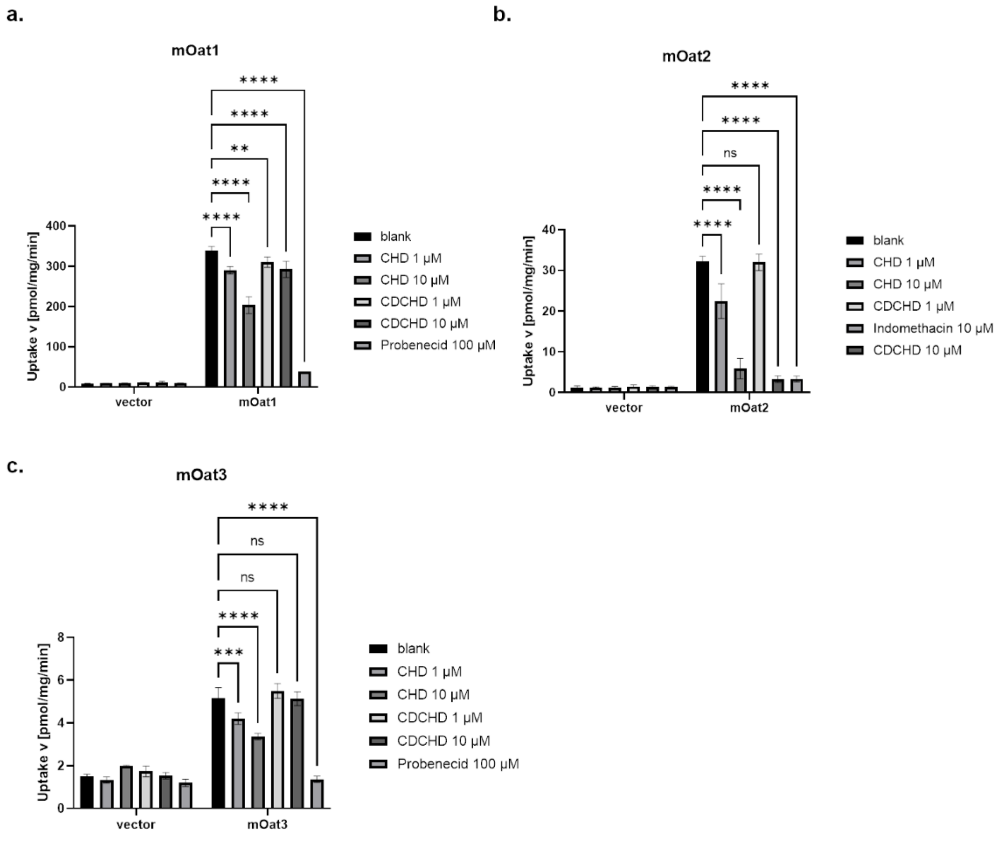

Figure S2. Uptake of substrate into mOat1-, mOat2- and mOat3-overexpressing cells upon addition of CHD and CDCHD.

Uptake of substrate into vector or mOat1- (a), mOat2- (b) and mOat3- (c) overexpressing cells. Cells were left untreated (black), were treated with 1 µM CHD (light grey), 10 µM CHD (medium-light grey), 1 µM CDCHD (very light grey), 10 µM CDCHD (dark grey) or inhibitor (medium-dark grey). For mOat1 (a) and mOat3 (c) 100 µM probenecid was used as inhibitor, whereas 10 µM indomethacin was used as inhibitor for mOat2 (b). n=3 per condition. Statistical testing was performed using an ordinary two-way ANOVA. ns: not significant; ** : $p < 0.01$; ***: $p < 0.001$; ****: $p < 0.0001$.

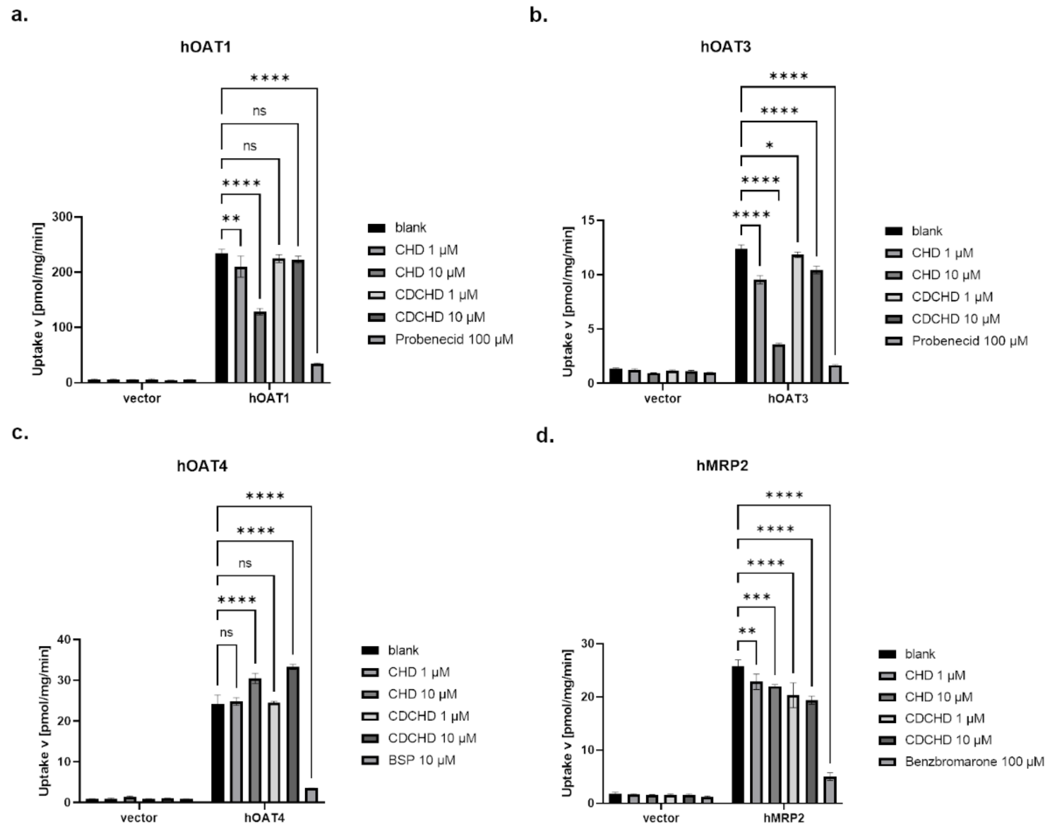

Figure S3. Uptake of substrate into hOAT1-, hOAT3-, hOAT4- and hMRP2-overexpressing cells upon addition of CHD and CDCHD.

Uptake of substrate into vector or hOAT1- (a), mOAT3- (b), hOAT4- (c) and hMRP2- (d) overexpressing cells. Cells were left untreated (black), were treated with 1 µM CHD (light grey), 10 µM CHD (medium-light grey), 1 µM CDCHD (very light grey), 10 µM CDCHD (dark grey) or inhibitor (medium-dark grey). For hOAT1 (a) and hOAT3 (c) 100 µM probenecid was used as inhibitor, whereas 10 µM BSP was used for hOAT4 (c) and 100 µM benzbromarone was used as inhibitor for hMRP2 (d). n=3 per condition. Statistical testing was performed using an ordinary two-way ANOVA. ns: not significant; *: $p < 0.05$; ** : $p < 0.01$; ***: $p < 0.001$; ****: $p < 0.0001$.

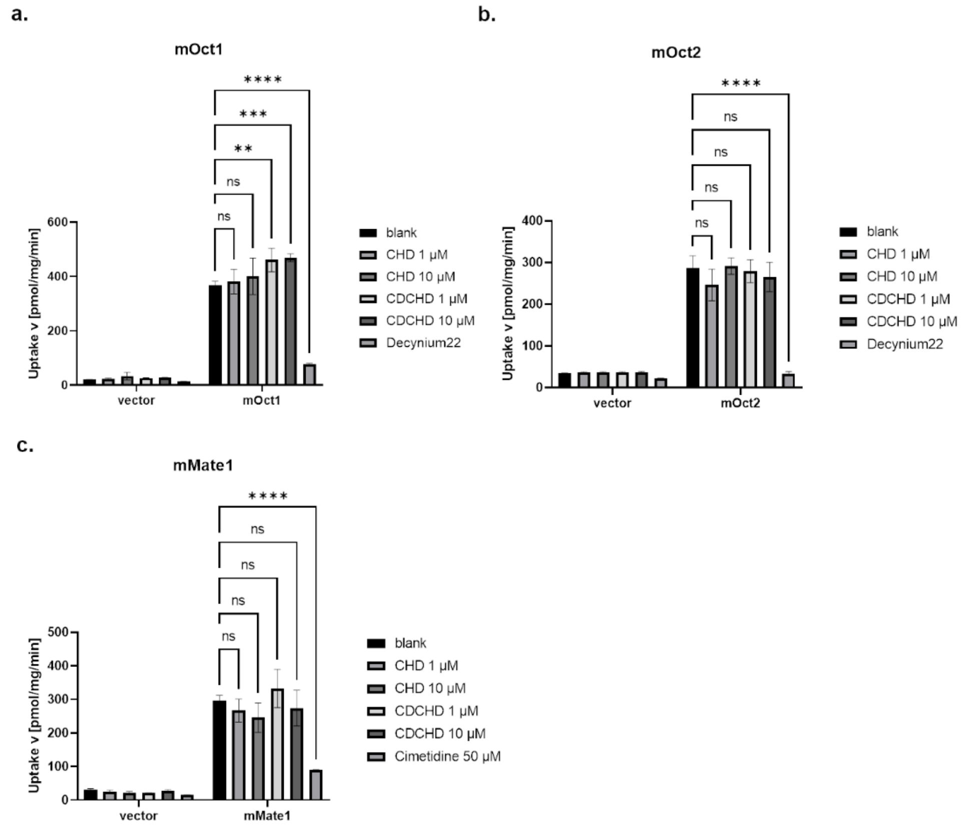

Figure S4. Uptake of substrate into mOct1-, mOct2- and mMate1-overexpressing cells upon addition of CHD and CDCHD.

Uptake of substrate into vector or mOct1- (a), mOct2- (b) and mMate1- (c) overexpressing cells. Cells were left untreated (black), were treated with 1 µM CHD (light grey), 10 µM CHD (medium-light grey), 1 µM CDCHD (very light grey), 10 µM CDCHD (dark grey) or inhibitor (medium-dark grey). For mOct1 (a) and mOct2 (b) decynium22 was used as inhibitor, whereas 50 µM cimetidine was used as inhibitor for mMate1 (c). n=3 per condition. Statistical testing was performed using an ordinary two-way ANOVA. ns: not significant; *: $p < 0.05$; ** : $p < 0.01$; ***: $p < 0.001$; ****: $p < 0.0001$.

a. b.

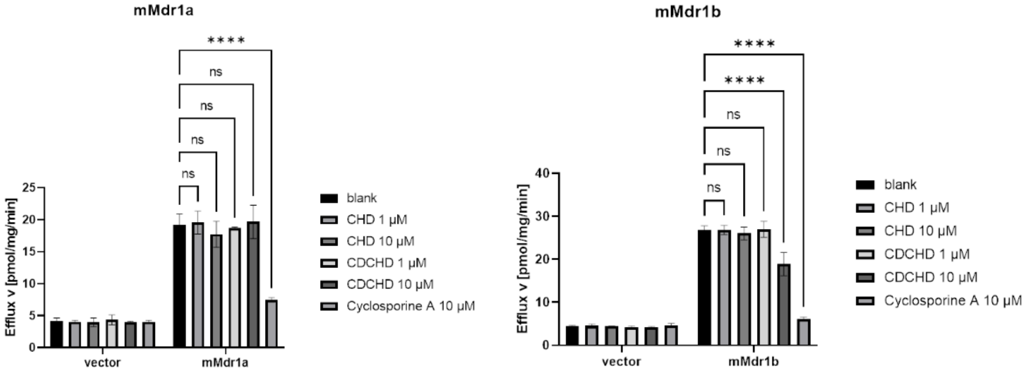

Figure S5. Efflux of substrate of mMdr1a- and mMdr1b-overexpressing cells upon addition of CHD and CDCHD.

Uptake of substrate into vector or mMdr1a- (a) and mMdr1b- (b) overexpressing cells. Cells were left untreated (black), were treated with 1 µM CHD (light grey), 10 µM CHD (medium-light grey), 1 µM CDCHD (very light grey), 10 µM CDCHD (dark grey) or inhibitor (medium-dark grey). Cyclosporine A (10 µM) was used as inhibitor (a,b). n=3 per condition. Statistical testing was performed using an ordinary two-way ANOVA. ns: not significant; ****: $p < 0.0001$.

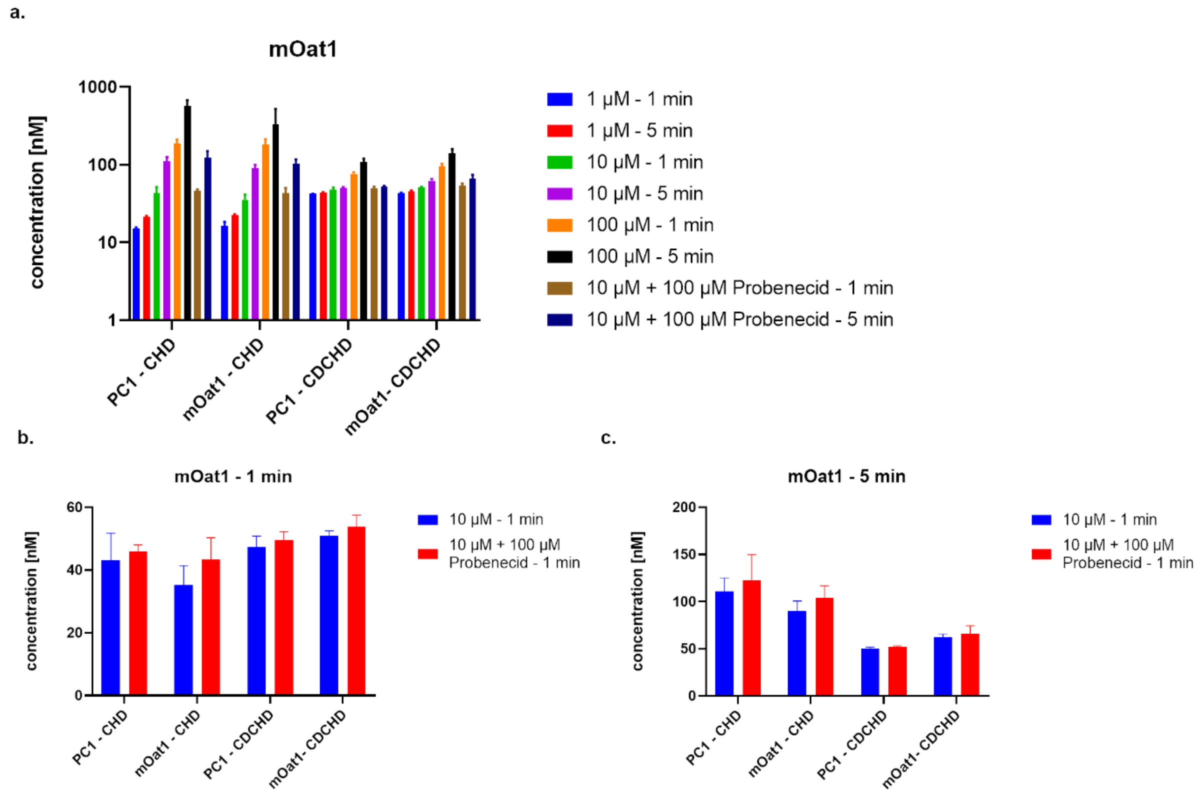

Figure S6. Intracellular concentrations of CHD and CDCHD in mOat1-overexpressing cells.

Intracellular concentrations of CHD and CDCHD in vector- (PC1) and mOat1-overexpressing cells (a-c). Cells were treated with either CHD or CDCHD at different concentrations and for different periods (a). Blue: 1 µM for 1 min; red: 1 µM for 5 min; green: 10 µM for 1 min; purple: 10 µM for 5 min; orange: 100 µM for 1 min, black: 100 µM for 1 min; brown: 10 µM + 100 µM probenecid for 1 min; dark blue: 10 µM + 100 µM probenecid for 5 min. Cells were treated with either CHD and CDCHD at 10 µM for 1 min (b) or for 5 min (c). Cells were either treated with CHD or CDCHD (blue) or with CHD or CDCHD and 100 µM probenecid (red) (b,c).

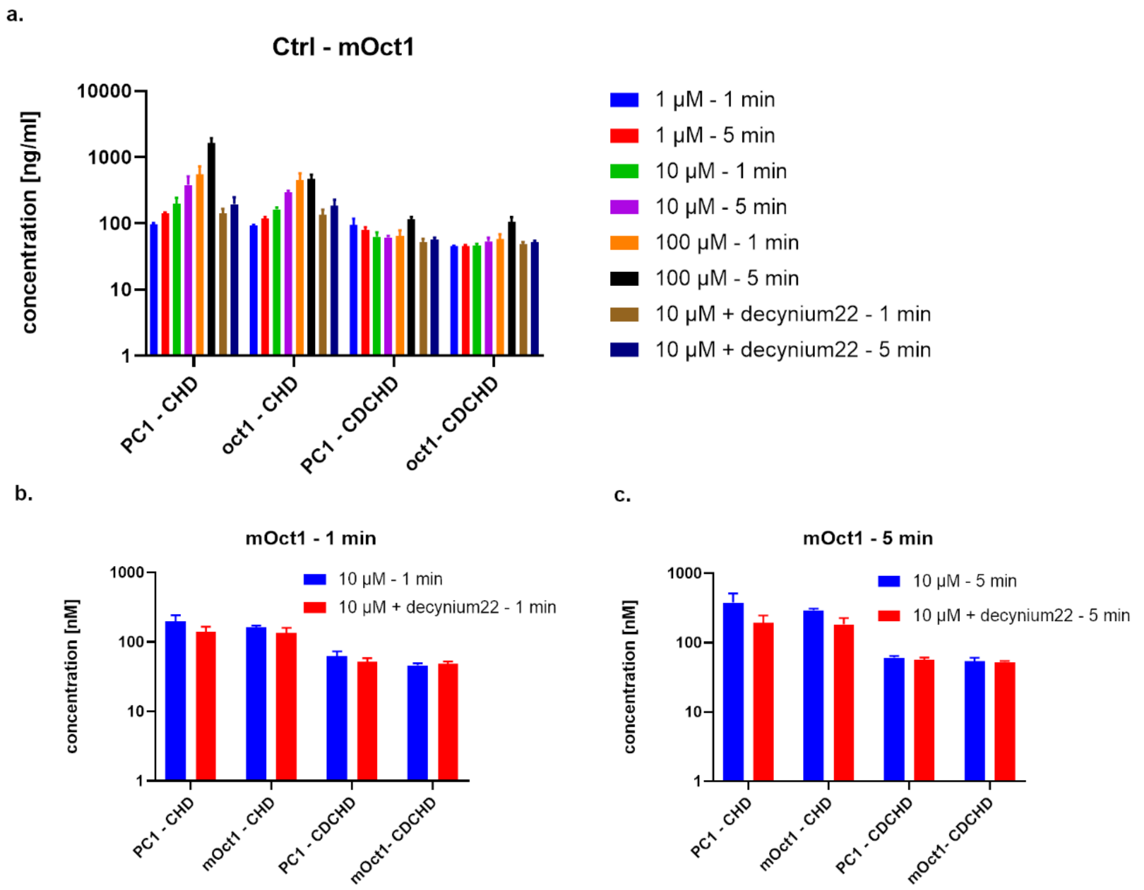

Figure S7. Intracellular concentrations of CHD and CDCHD in mOct1-overexpressing cells.

Intracellular concentrations of CHD and CDCHD in vector- (PC1) and mOct1-overexpressing cells (a-c). Cells were treated with either CHD or CDCHD at different concentrations and for different periods (a). Blue: 1 µM for 1 min; red: 1 µM for 5 min; green: 10 µM for 1 min; purple: 10 µM for 5 min; orange: 100 µM for 1 min, black: 100 µM for 1 min; brown: 10 µM + decynium22 for 1 min; dark blue: 10 µM + decynium22 for 5 min. Cells were treated with either CHD and CDCHD at 10 µM for 1 min (b) or for 5 min (c). Cells were either treated with CHD or CDCHD (blue) or with CHD or CDCHD and decynium22 (red) (b,c).

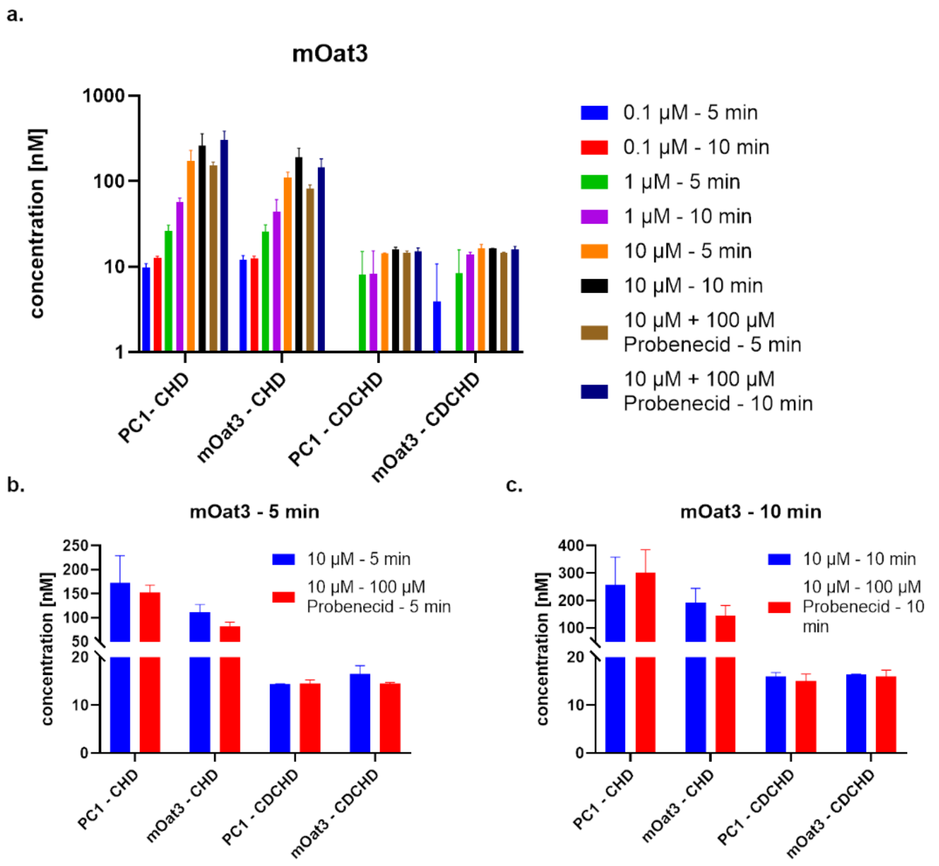

Figure S8. Intracellular concentrations of CHD and CDCHD in mOat3-overexpressing cells.

Intracellular concentrations of CHD and CDCHD in vector- (PC1) and mOat3-overexpressing cells (a-c). Cells were treated with either CHD or CDCHD at different concentrations and for different periods (a). Blue: 0.1 µM for 5 min; red: 0.1 µM for 10 min; green: 1 µM for 5 min; purple: 1 µM for 10 min; orange: 10 µM for 5 min, black: 10 µM for 5 min; brown: 10 µM + 100 µM probenecid for 1 min; dark blue: 10 µM + 100 µM probenecid for 5 min. Cells were treated with either CHD and CDCHD at 10 µM for 5 min (b) or for 10 min (c). Cells were either treated with CHD or CDCHD (blue) or with CHD or CDCHD and 100 µM probenecid (red) (b,c).

Supporting tables

Table S1. Inhibitors as well as substrates including their concentrations per transporter.

Transporter	Substrate	Inhibitor
**mOat1**	PAH [10 µM]	probenecid [100 µM]
**mOat2**	cGMP [10 µM]	Indomethacin [10 µM]
**mOat3**	estrone-3-sulfate [1 µM]	probenecid [100 µM]
**hOAT1**	PAH [10 µM]	probenecid [100 µM]
**hOAT3**	estrone-3-sulfate [1 µM]	probenecid [100 µM]
**hOAT4**	estrone-3-sulfate [1 µM]	BSP [10 µM]
**hMRP2**	CDCF-DA [5 µM]	benzbromarone [100 µM]
**mOct1**	MPP [10 µM]	decynium22 [50 µM]
**mOct2**	MPP [10 µM]	decynium22 [50 µM]
**mMate1**	metformin [20 µM]	cimetidine [50 µM]
**mMdr1a**	rhodamin123 [10 µM]	cyclosporine A [10 µM]
**mMdr1b**	rhodamin123 [10 µM]	cyclosporine A [10 µM]

Table S2. Mass transitions for CHD and CDCHD using caffeine as internal standard

	Q1 mass	*Q3 mass*	*DP [volts]*	*CE [volts]*	*CXP [volts]*
Caffeine	195.024	138.0	130	25	14
		110.0	130	31	18
CDCHD	413.073	396.0	130	21	22
		378.0	130	23	20
CHD	412.056	271.0	130	21	14
		253.1	130	33	16

DP: declustering potential; CE: collision energy; CXP: cell exit potential
